# Facile Synthesis of Weakly Ferromagnetic Organogadolinium Macrochelates‐Based T_1_‐Weighted Magnetic Resonance Imaging Contrast Agents

**DOI:** 10.1002/advs.202205109

**Published:** 2022-11-15

**Authors:** Yudie Lu, Zhiyu Liang, Jie Feng, Lin Huang, Shuai Guo, Peiwei Yi, Wei Xiong, Sijin Chen, Sugeun Yang, Yikai Xu, Yan Li, Xiaoyuan Chen, Zheyu Shen

**Affiliations:** ^1^ School of Biomedical Engineering Southern Medical University Guangzhou Guangdong 510515 China; ^2^ Medical Imaging Center Nanfang Hospital Southern Medical University Guangzhou Guangdong 510515 China; ^3^ Department of Biomedical Science BK21 FOUR Program in Biomedical Science and Engineering Inha University College of Medicine Incheon 22212 South Korea; ^4^ Departments of Diagnostic Radiology, Surgery, Chemical and Biomolecular Engineering, and Biomedical Engineering Clinical Imaging Research Centre Nanomedicine Translational Research Program Yong Loo Lin School of Medicine and Faculty of Engineering National University of Singapore Singapore 119228 Singapore

**Keywords:** hundred‐gram‐scale facile synthesis, magnetic resonance imaging (MRI) contrast agents (CAs), organogadolinium macrochelates (OGMCs), outstanding relaxivities, weak ferromagnetism

## Abstract

To surmount the major concerns of commercial small molecule Gd chelates and reported Gd‐based contrast agents (GBCAs) for magnetic resonance imaging (MRI), a new concept of organogadolinium macrochelates (OGMCs) constructed from the coordination between Gd^3+^ and macromolecules is proposed. A library of macromolecules were screened for Gd^3+^ coordination, and two candidates [i.e., poly(acrylic acid) (PAA), and poly(aspartic acid) (PASP)] succeeded in OGMC formation. Under optimized synthesis conditions, both Gd‐PAA12 and Gd‐PASP11 OGMCs are outstanding *T*_1_‐weighted CAs owing to their super high *r*_1_ values (> 50 mm ^−1^ s^−1^, 3.0 T) and ultralow *r*_2_/*r*_1_ ratios (< 1.6, 3.0 T). The ferromagnetism of OGMCs is completely different from the paramagnetism of commercial and reported GBCAs. The ferromagnetism is very weak (M_s_ < 1.0 emu g^−1^) leading to a low *r*_2_, which is preferred for *T*_1_ MRI. Gd^3+^ is not released from the OGMC Gd‐PAA12 and Gd‐PASP11, ensuring biosafety for in vivo applications. The safety and *T*_1_‐weighted MRI efficiencies of the OGMC Gd‐PAA12 and Gd‐PASP11 are tested in cells and mice. The synthesis method of the OGMCs is facile and easy to be scaled up. Consequently, the OGMC Gd‐PAA12 and Gd‐PASP11 are superior *T*_1_‐weighted CAs with promising translatability to replace the commercial Gd chelates.

## Graphical abstract

To overcome the problems of commercial small molecule Gd chelates and reported Gd‐based contrast agents (GBCAs) for *T*_1_‐weighted magnetic resonance imaging (MRI), a new concept of organogadolinium macrochelates (OGMCs) constructed from the coordination between Gd^3+^ and macromolecules is proposed in this study, and a hundred‐gram‐scale facile synthesis method is developed to prepare two weakly ferromagnetic OGMCs with outstanding relaxivities for *T*_1_‐weighted MRI.

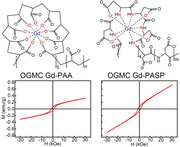

## 1 Introduction

Magnetic resonance imaging (MRI) has been widely used in clinical practice with advantages of non‐invasiveness, non‐radiation damage, excellent soft‐tissue contrast, high spatial resolution, and arbitrary azimuth tomography imaging.^[^ ^1^ ^]^ Contrast agents (CAs) are preferentially prescribed to enhance the MRI quality for certain diseases,^[^ ^2^ ^]^ including *T*_1_‐weighted CAs with positive (i.e., bright) signals and *T*_2_‐weighted CAs with negative (i.e., dark) signals.^[^ ^3^ ^]^ Magnetic iron oxide nanoparticles (MIONs) as *T*_2_‐weighted CAs have quit the market due to the instrinsic issues of dark signals, slow body clearance, susceptibility artifacts resulted from the high magnetic moment, long waiting time for patients due to the long blood circulation, long processing time for the busy clinical MRI machines because of the long repetition time (TR) and echo time (TE).^[^ ^4^ ^]^ Therefore, the *T*_1_‐weighted CAs have been occupying almost the whole market of MRI CAs.^[^ ^5^ ^]^

Although the reported exceedingly small MIONs (ES‐MIONs) are widely studied as *T*_1_‐weighted CAs,^[^ ^6^ ^]^ the current commercial *T*_1_‐weighted CAs are all small molecule gadolinium (Gd) chelates, including Magnevist (Gd‐DTPA, approved in 1988), Eovist (Gd‐EOB‐DTPA, approved in 2008) and Gadavist (Gd‐DO3A‐Butriol, approved in 2011) produced by Bayer Schering Pharma, Germany, ProHance (Gd‐DO3A‐HP, approved in 1992) and Multihance (Gd‐BOPTA, approved in 2004) produced by Bracco Imaging, Italy, OptiMARK (Gd‐DTPA‐BMEA, approved in 1999) produced by Mallinckrodt Inc, USA, Omniscan (Gd‐DTPA‐BMA, approved in 1993) produced by Amersham‐Nycomed, Norway, and Dotarem (Gd‐DOTA, approved in 2013) produced by Guerbet SA, France.^[^ ^7^ ^]^ However, because the *r*_1_ values of these Gd chelates are rather low (≈4 mm ^−1^ s^−1^), the clinical dosages are as high as 15.7 mg Gd kg^−1^ body weight, resulting in potential risks of nephrotoxicity (e.g., nephrogenic systemic fibrosis) and Gd deposition in the brain, which have been warned by the U.S. food and drug administration (FDA).^[^ ^8^ ^]^ Therefore, enhancing *r*_1_ value is considered to be an important strategy for designing new Gd‐based CAs (GBCAs) with low clinical dosages, reduced nephrotoxicity and Gd deposition in the brain.

According to the classical relaxation model and Solomon‐Bloembergen‐Morgan (SBM) theory, the *T*_1_ relaxation rate can be predicted from equations (1), (2) and (3).^[^ ^9^ ^]^

(1) $$\frac{1}{\tau_{\mathrm{Ci}}}=\frac{1}{\tau_{R}}+\frac{1}{T_{\mathrm{ie}}}+\frac{1}{\tau_{M}}$$

(2) $$\frac{1}{T_{1m}}=\frac{2}{15}\frac{\gamma^{2}g^{2}S\left(S+1\right)\mu_{B}^{2}}{r_{\mathrm{Gd}-H}^{6}}\left(\frac{3\tau_{C1}}{1+\omega_{H}^{2}\tau_{C1}^{2}}+\frac{7\tau_{C2}}{1+\omega_{S}^{2}\tau_{C2}^{2}}\right)$$

(3) $$\frac{1}{T_{1}}=\frac{qP_{m}}{T_{1m}+\tau_{M}}$$

Equation (1) indicates the modulation of the correlation time *τ*_Ci_ (*i* = 1, 2) describing the fluctuating magnetic dipole. Where, *τ*_R_ is the rotational correlation time of the contrast agent; *T*_ie_ is the electron relaxation time; *τ*_M_ is the mean water residence time. *T*_1m_ is the *T*_1_ relaxation time of the water proton, as defined by equation (2). *γ*, g, S, and *r*_Gd‐H_ are the gyromagnetic of the proton, the electronic g‐factor, the spin‐quantum number of the corresponding paramagnetic matter, and the distance between the contrast center and the proton, respectively. In Equation (3), 1/*T*_1_ is the longitudinal relaxation rate, *q* is the number of a bound water molecule, and *P*_m_ is the mole fraction of water coordinated to the metal center. In theory, improving the 1/*T*_1_ of the contrast medium mainly requires maximizing *q* and optimizing *τ*_M_, *τ*_R_ and *T*_ie_.^[^ ^4^, ^10^ ^]^

The main reason for the low *r*_1_ values of the commercial Gd‐chelates is the fast tumbling in water due to their small molecular weights because a small *τ*_R_ value (i.e., tumbling time) can lead to a low *r*_1_ value according to the equations (1), (2) and (3).

Although the small *q* and *P*_m_ values of the reported Gd oxide nanoparticles (GONs), which are both smaller than those of the commercial Gd‐chelates, are detrimental to *r*_1_ values in accordance with equation (3). The reported *r*_1_ values of GONs are much higher than the commercial Gd‐chelates^[^ ^11^ ^]^ because the GONs have much higher *τ*_R_ values due to the larger sizes. The dosage of GONs can be reduced due to the high *r*_1_ values, but the potential risks of nephrotoxicity and Gd deposition in the brain are not mitigated as the GONs are degradable in acidic lysosomes to release toxic Gd^3+^ after endocytosis.^[^ ^12^ ^]^

To surmount the problems of commercial Gd‐chelates and reported GONs, in this study, we propose a new concept of organogadolinium macrochelates (OGMCs), which are constructed from the coordination between Gd^3+^ and macromolecules. The high *τ*_R_ values of OGMCs can result in high *r*_1_ values, and the strong coordination between Gd^3+^ and macromolecules can avoid the Gd^3+^ release in vivo.

**Figure 1:**
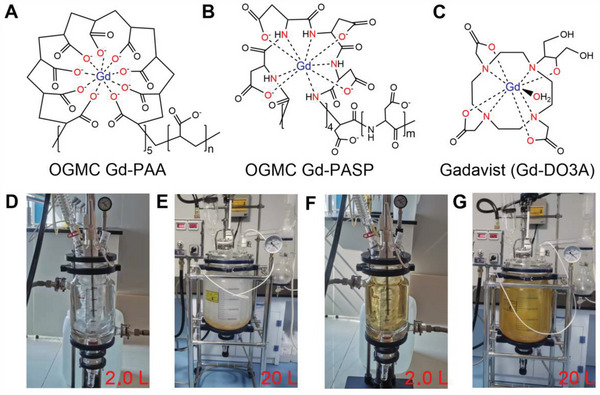
Structural formula and large‐scale synthesis of the OGMCs. A–C) Structural formula of the OGMC Gd‐PAA A), Gd‐PASP B), and commercial Gadavist C). D–G) Photographs of 2.0 L D,F) or 20 L E,G) of reactors for large‐scale synthesis of the OGMC Gd‐PAA D,E), or Gd‐PASP F,G).

To verify the practicability of the OGMC concept, in this study, a number of macromolecules were screened for Gd^3+^ coordination and two candidates [i.e., poly(acrylic acid) (PAA), and poly(aspartic acid) (PASP)] succeeded in OGMC formation, whose structural formulas are respectively shown in **Figure** **1**A,B compared with the structure of the commercial GBCA Gadavist (Figure 1C). Typically, based on coordination chemistry, Gd^3+^ reacts with the carboxyl groups of PAA to generate OGMC Gd‐PAA, or chelate with the carboxyl and amino groups of PASP to produce OGMC Gd‐PASP. Both Gd‐PAA and Gd‐PASP are outstanding *T*_1_‐weighted CAs owing to their super high *r*_1_ values (> 50 mm ^−1^ s^−1^, 3.0 T) and ultralow *r*_2_/*r*_1_ ratios (< 1.6, 3.0 T). In addition, their very weak ferromagnetism is completely different from the paramagnetism of the commercial and reported GBCAs, which demonstrates that our ferromagnetic OGMCs are brand new GBCAs. More importantly, the synthesis method of the OGMCs is facile and easy to be scaled up (Figure 1D–G).

## 2 Results and Discussion

### 2.1 Synthesis and Characterization of the Organogadolinium Macrochelates (OGMCs)

The OGMC Gd‐PAA and Gd‐PASP with simple components were synthesized via the coordination reaction between Gd^3+^ and carboxyl and/or amino groups of macromolecules (Figure 1A,B). The synthesis conditions and characterization results of the OGMC Gd‐PAA and Gd‐PASP synthesized with different molecular weights (M_w_) of PAA and PASP are shown in Table S1 (Supporting Information). The M_w_ of PAA and PASP was respectively optimized to be 5100 and 7500 due to the higher Gd recoveries (94.9%, 93.0%) and the higher *r*_1_ values (49.80 ± 0.88 mm ^−1^ s^−1^, 49.77 ± 0.79 mm ^−1^ s^−1^, 3.0 T) with comparable *r*_2_/*r*_1_ ratios (1.62 ± 0.08, 1.31 ± 0.07, 3.0 T). Compared with other pH values, pH 10 was chosen as the optimum for the synthesis of the OGMC Gd‐PAA and Gd‐PASP based on the higher *r*_1_ values (Tables S2 and S3, Figure S1A,B, Supporting Information). The slightly alkaline condition benefits the coordination between Gd^3+^ and carboxyl groups, but strongly alkaline condition causes the formation of ionic bond between Gd^3+^ and carboxyl groups. Furthermore, the polymer/Gd mass ratio was respectively optimized to be 20.35 and 10.17 for the synthesis of Gd‐PAA10 (*r*_1_ = 55.01 ± 0.55 mm ^−1^ s^−1^) and Gd‐PASP6 (*r*_1_ = 53.68 ± 0.76 mm ^−1^ s^−1^) due to the higher *r*_1_ values and similar *r*_2_/*r*_1_ ratios (Tables S2 and S3, Figure S1C,D, Supporting Information).

In addition, the influence of the reaction temperature and nitrogen atmosphere on the MRI capability of Gd‐PAA or Gd‐PASP were further explored. Figures S2–S4 (Supporting Information) show the linear fitting plots of *T*_1_ relaxation rate (1/*T*_1_) or *T*_2_ relaxation rate (1/*T*_2_) as a function of Gd concentration (*C*_Gd_) for Gd‐PAA12‐15, Gd‐PASP11‐14, Gadavist, Magnevist, and Gd(NO_3_)_3_ at 3.0 T and 7.0 T. The *r*_1_ and *r*_2_ values obtained from the slopes are summarized in Table S4 (Supporting Information). The R^2^ values of the fitted lines are all very close to 1.00, indicating the strong concentration gradient dependence of MRI signal. Both 3.0 T and 7.0 T MRI results show that the *r*_1_ value and *r*_2_/*r*_1_ ratio of Gd‐PAA12‐15 or Gd‐PASP11‐14 differ slightly (Table S4, Supporting Information), indicating that the reaction temperature and nitrogen atmosphere protection have little influence on the chelation of Gd^3+^ with PAA or PASP. The *r*_1_ values at 3.0 T of the optimal samples Gd‐PAA12 and Gd‐PASP11 are 56.23 ± 1.69 mm ^−1^ s^−1^ and 54.00 ± 1.47 mm ^−1^ s^−1^, respectively. The corresponding *r*_2_/*r*_1_ ratios are 1.54 ± 0.03 and 1.49 ± 0.02, respectively. The super high *r*_1_ values can be attributed to the steric effect of macromolecules of PAA and PASP, which limits the tumbling of Gd, resulting in long *τ*_R_ of the OGMCs.^[^ ^13^ ^]^ Compared with the Gd(NO_3_)_3_ solution and the commercial MRI CAs of Gadavist and Magnevist (Table S4, Supporting Information), the enormously increased *r*_1_ values demonstrate the much stronger *T*_1_‐MRI capability of our OGMCs. The much stronger T_1_‐MRI capability indicates that the Gd dosage for clinical MRI could be significantly decreased, resulting in lower risks of nephrotoxicity (e.g., nephrogenic systemic fibrosis) and Gd deposition in the brain. Therefore, our OGMCs are promising to solve the problems caused by other reported GBCAs.

**Figure 2:**
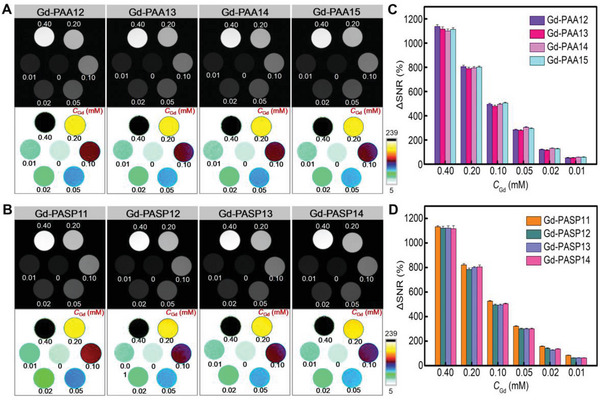
*T*_1_‐weighted MR imaging of the OGMCs at various concentrations. A,B) The black & white and corresponding pseudo‐color images of *T*_1_‐weighted MR images for Gd‐PAA12‐15 A), or Gd‐PASP11‐14 B) observed by a 7.0 T MRI scanner. C,D) ΔSNR of the MR images for Gd‐PAA12‐15 A), or Gd‐PASP11‐14 B) with various *C*_Gd_ (*n* = 3).

**Figure 3:**
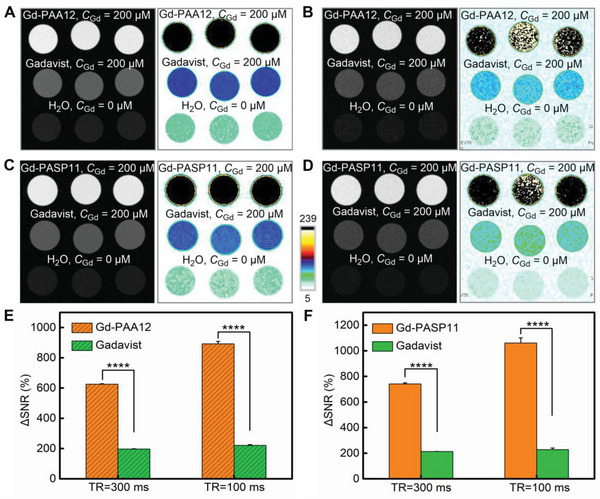
*T*_1_‐weighted MRI performance of the OGMCs compared with Gadavist and pure water. A–D) *T*_1_‐weighted MR images of Gd‐PAA12 A,B), or Gd‐PASP11 C,D) compared with Gadavist and pure water with 300 ms A,C), or 100 ms B,D) of repetition time (TR). Echo time (TE) = 7.3 ms. Magnetic field = 7.0 T. *C*_Gd_ = 200 µm. E,F) ΔSNR of the MR images for Gd‐PAA12 E), or Gd‐PASP11 F) compared with Gadavist. ****P < 0.0001.

To evaluate the feasibility of the OGMC Gd‐PAA and Gd‐PASP for MR imaging, the *T*_1_‐weighted images of Gd‐PAA12‐15 and Gd‐PASP11‐14 solutions were performed on a 3.0 or 7.0 T MRI scanner. The MR image brightness of commercial Gadavist or Magnevist with 0.40 mm of *C*_Gd_ are even lower than that of Gd‐PAA12‐15 or Gd‐PASP11‐14 with 0.10 mm of *C*_Gd_ (Figure S5A,B, Supporting Information). In addition, the MR image brightness of Gd‐PAA12‐15 or Gd‐PASP11‐14 increased with the increasing *C*_Gd_ from 0.01 to 0.40 mm, at both 3.0 T and 7.0 T (**Figure** **2**A,B; Figure S5A,B, Supporting Information). The ΔSNR values of 3.0 and 7.0 T MR images for Gd‐PAA12‐15 and Gd‐PASP11‐14 with various *C*_Gd_ (Figure 2C,D; Figure S5C,D, Supporting Information) manifest the concentration gradient dependence of MRI signal, indicating the strong *T*_1_‐weighted imaging performance. **Figure** **3**A–D show the *T*_1_‐weighted MR images of Gadavist, Gd‐PAA12 and Gd‐PASP11 solutions at the same *C*_Gd_ (200 µm) using pure water as a control. The grey scale and corresponding pseudo‐color images clearly show that the MRI effect of both Gd‐PAA12 and Gd‐PASP11 outperform commercial Gadavist. The quantitative results indicate significant differences in contrast enhancement (ΔSNR) of Gd‐PAA12 or Gd‐PASP11 compared to Gadavist (****P < 0.0001, Figure 3E,F). These results further demonstrate that Gd‐PAA12 and Gd‐PASP11 are potential candidates for practical applications as *T*_1_‐weighted CAs.

**Figure 4:**
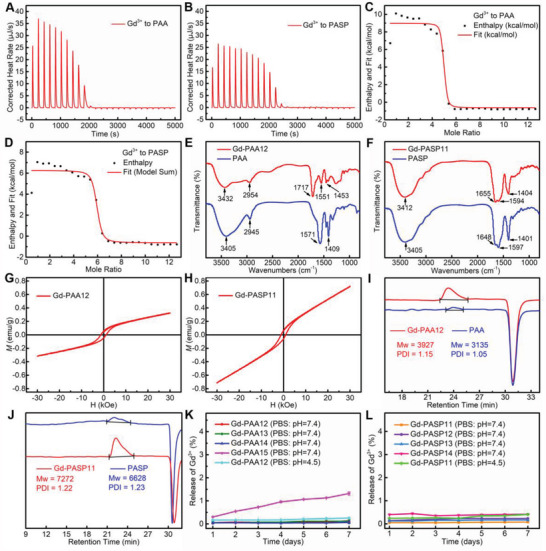
Characterization of the OGMCs. A–D) The raw thermogram A,B) and binding isotherm C,D) obtained in the isothermal titration calorimetry (ITC) analysis for the interaction between Gd^3+^ and PAA A,C), or PASP B,D). E,F) FT‐IR spectra of PAA and Gd‐PAA12 E), or PASP and Gd‐PASP11 F). G,H) Field‐dependent magnetization curves of Gd‐PAA12 G), or Gd‐PASP11 H) measured by physical property measurement system (PPMS) at 298 K. I,J) GPC chromatogram of PAA and Gd‐PAA12 I), or PASP and Gd‐PASP11 J). K, L) The time‐dependent release of free Gd^3+^ from Gd‐PAA12‐15 K) or Gd‐PASP11‐14 L) in PBS at pH 7.4 or 4.5.

The heat exchange in the interaction between Gd^3+^ and PAA or PASP was measured by an isothermal titration calorimeter (ITC) to clarify the mechanism from the perspective of thermodynamics. The titration curves (**Figure** **4**A–D) show that Gd^3+^ does interact with PAA or PASP, and the equilibrium dissociation constant (Kd) of Gd‐PAA (Kd = 2.26 × 10^−7^ m) is close to that of Gd‐PASP (Kd = 6.89 × 10^−7^ m) (Table S5, Supporting Information). Both Kd values are very small, indicating that there is a strong binding affinity between Gd^3+^and PAA or PASP. Moreover, the formation of Gd‐PAA or Gd‐PASP is accompanied by positive ΔS and ΔH values of the endothermic reaction, which is driven by entropy and can proceed spontaneously.^[^ ^14^ ^]^ By ITC fitting, the stoichiometric ratios of Gd^3+^ to PAA and PASP are 4.7 and 5.7, respectively (Table S5, Supporting Information), from which the theoretical Gd loading contents (i.e., the mass percentage of the loaded Gd to PAA or PASP) should be 12.7% and 10.8% for Gd‐PAA and Gd‐PASP, respectively. After optimization of the synthesis conditions, the real Gd loading contents in Gd‐PAA12 and Gd‐PASP11 were measured to be 5.4% and 10.5%. There is a remarkable difference between the theoretical Gd loading content and the actual value for Gd‐PAA12, but only a little difference for Gd‐PASP11. That's because the feeding concentrations of Gd^3+^ were respectively 62.5 and 125 mm for the synthesis of Gd‐PAA12 and Gd‐PASP11. The measured contents are lower than the theoretical Gd loading contents, indicating that the carboxyl and/or amino groups in Gd‐PAA and Gd‐PASP are superfluous to coordinate with Gd^3+^. Once Gd^3+^ is free out, excess carboxyl and/or amino groups will quickly capture and coordinate with the free Gd^3+^. Because the coordination of the carboxyl group of Gd‐PAA12 and the carboxyl and amino groups of Gd‐PASP11 with Gd^3+^ is firm, free Gd^3+^ can hardly leak from the OGMC Gd‐PAA or Gd‐PASP, thus enhancing the biosafety for in vivo applications.

Fourier transform infrared spectroscopy (FT‐IR) was used to verify the composition and functional group changes of the OGMCs. In the FT‐IR spectrum of Gd‐PAA12 (Figure 4E), 1551 and 1453 cm^−1^ are the antisymmetric and symmetric stretching vibration absorption peaks of −COO^−^, which are slightly different from those of the PAA (1571 and 1409 cm^−1^). In addition, a new C = O stretching vibration absorption peak in carboxyl groups appears at 1717 cm^−1^ for Gd‐PAA12. These results indicate that oxygen atoms in the carboxyl group of PAA is coordinated with Gd^3+^.^[^ ^15^ ^]^ Regarding the FT‐IR spectrum of Gd‐PASP11 compared with that of the PASP (Figure 4F), the peak at 1648 cm^−1^ moves to a high wavenumber of 1655 cm^−1^, which is attributed to the stretching vibration absorption of C = O and the in‐plane deformation vibration of N‐H. The stretching vibration peaks of O‐H in hydroxyl groups and N‐H in amide groups also shift from 3405 to 3412 cm^−1^. These results suggest that the chelation of Gd^3+^ affects the peaks in FT‐IR spectrum of Gd‐PASP11, and the amino and carboxyl groups of PASP are involved in coordination with Gd^3+^.

The field‐dependent magnetization curves show that both Gd‐PAA12 (Figure 4G) and Gd‐PASP11 (Figure 4H) are weakly ferromagnetic, whose coercivities are 1.31 and 1.14 kOe, and saturation magnetization (M_s_) values are 0.33 and 0.72 emu g^−1^ at 30 kOe. The ferromagnetism of the OGMC Gd‐PAA12 and Gd‐PASP11 is completely different from the paramagnetism of commercial and reported GBCAs,^[^ ^16^ ^]^ which demonstrates that our ferromagnetic OGMCs are brand new GBCAs. It is well‐known that the ferromagnetism is usually much stronger (with M_s_ up to dozens or hundreds emu/g) than the paramagnetism,^[^ ^17^ ^]^ and results in a large *r*_2_ value according to the equation (4). The very weak ferromagnetism like our OGMCs (M_s_ < 1.0 emu g^−1^) is rarely reported and leads to a low *r*_2_ value, which is good for *T*_1_‐weighted MRI.

(4) $$\frac{1}{T_{2}}=\frac{\left(256\pi^{2}\gamma^{2}/405\right)V^{*}M_{s}^{2}r^{2}}{D\left(1+L/r\right)}$$

Because both normal cells and tumor cells are negatively charged, positively charged nanomaterials are easy to break the membrane structures of normal cells before encountering tumor cells, showing cytotoxicity. Our Gd‐PAA12 and Gd‐PASP11 are both negatively charged, whose zeta potentials were measured to be −12.64 ± 2.94 mV, and −3.01 ± 0.36 mV, respectively (Figure S6, Supporting Information). Therefore, our organogadolinium macrochelates show no cytotoxicity in vivo before passive targeting to tumors.

The M_w_ of Gd‐PAA12 and PAA measured by a gel permeation chromatograph (GPC) system are 3927 and 3135 (Figure 4I), indicating that one PAA macromolecule coordinates with five Gd^3+^ generating the macrochelate of Gd‐PAA12. In addition, the GPC results also show that the M_w_ of Gd‐PASP11 and PASP are 7272 and 6628 (Figure 4J), which demonstrates that the macrochelate of Gd‐PASP11 includes one PASP macromolecule and four Gd^3+^. Furthermore, the similar PDI values for Gd‐PAA12 (1.15) compared with PAA (1.05), and Gd‐PASP11 (1.22) compared with PASP (1.23) suggest the narrow M_w_ distributions and controllable synthesis for Gd‐PAA12 and Gd‐PASP11.^[^ ^18^ ^]^

It is well‐known that gadolinium is easily coordinated with O, N, especially with the carboxyl group, and the coordination number of nine is preferred for lanthanide cation Gd^3+^. All commercial contrast agents are strictly nine coordinated with organic ligands.^[^ ^9a^ ^]^ The results in Figure 4K,L shows that the released Gd^3+^ is < 2.0% for the OGMC Gd‐PAA15, and even < 0.50% for Gd‐PAA12‐14 and Gd‐PASP11‐14 within one week in PBS at pH 7.4 or 4.5, indicating that Gd^3+^ almost cannot be released from the OGMC Gd‐PAA and Gd‐PASP. That is because the carboxyl groups of Gd‐PAA and the carboxyl and amino groups of Gd‐PASP are excess to coordinate with Gd^3+^ by nine coordination bonds, and the coordination is firm,^[^ ^19^ ^]^ which is double insurance against Gd^3+^ release ensuring the biosafety for in vivo applications.

Figure S7 (Supporting Information) shows the stability tests under worse conditions (highly acidic, higher temperatures, or in the presence of Zn^2+^ competition). When incubated in PBS with pH 2.0 at 37 °C, up to 35% of Gd^3+^ is released from Gd‐PAA12 within one week, indicating that Gd‐PAA12 is unstable under highly acidic conditions. While Gd‐PASP11 is relatively stable at pH 2.0, the released Gd^3+^ is < 3%. In PBS with pH 7.4 at 60 °C, the released Gd^3+^ is < 2% for Gd‐PAA12 and Gd‐PASP11, showing their strong stability at the higher temperature. Furthermore, the stability of Gd‐PAA12 and Gd‐PASP11 was studied in transmetallation experiments against Zn^2+^, which is a major potential competitor for the displacement of Gd^3+^.^[^ ^20^ ^]^ In PBS with pH 7.4 at 37 °C, Gd‐PAA12 or Gd‐PASP11 was directly mixed with a 2‐fold concentration of Zn^2+^ in the dialysis bag, and the released Gd^3+^ is less than 2.0% for Gd‐PAA12 and Gd‐PASP11. Therefore, these experiments directly indicate the high stability of our OGMC Gd‐PAA12 and Gd‐PASP11.

### 2.2 Evaluation of Effectiveness and Safety on Cells

**Figure 5:**
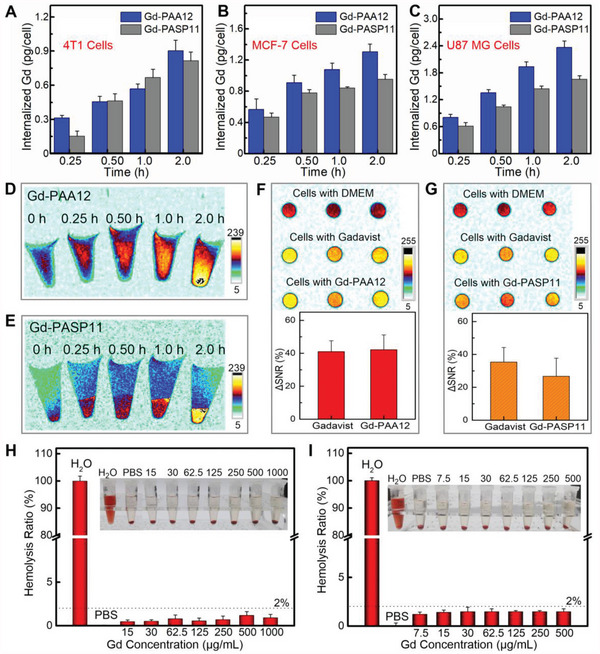
Efficacy and safety evaluation on cells. A–C) The internalized amount of Gd‐PAA12 and Gd‐PASP11 into 4T1 A), MCF‐7 B), or U87 MG C) cells for various durations (0.25, 0.50, 1.0, or 2.0 h). D,E) *T*_1_‐weighted MR images of MCF‐7 cell pellets (slice orientation: sagittal) incubated with Gd‐PAA12 D), or Gd‐PASP11 E) at various incubation time. F,G) *T*_1_‐weighted MR images and ΔSNR of MCF‐7 cell pellets (slice orientation: axial) incubated with Gd‐PAA12 F), or Gd‐PASP11 G) for 2.0 h compared with DMEM and Gadavist (7.0 T). H,I) Hemolysis ratio induced by Gd‐PAA12 H) or Gd‐PASP11 I) at various *C*_Gd_. Mean ± SD, *n* = 3.

The confocal laser scanning microscope (CLSM) images of human normal hepatocytes (LO2, as the control) and tumor cells (MCF‐7 and 4T1 cells) incubated with Rhodamine 6G (R6G) labeled Gd‐PAA12 or Gd‐PASP11 are shown in Figures S8 and S9 (Supporting Information). Much red fluorescence can be found inside the cell membrane after incubation, indicating the effective uptake of the R6G‐labeled Gd‐PAA12 or Gd‐PASP11 by LO2, MCF‐7 or 4T1 cells. The cellular uptakes of Gd‐PAA12 and Gd‐PASP11 by 4T1, MCF‐7 and U87 MG cells were then quantified by ICP‐MS (**Figure** **5**A–C). The internalized Gd amounts in 4T1, MCF‐7 and U87 MG cells after 2.0 h incubation were measured to be 0.90 ± 0.09, 1.31 ± 0.10, and 2.37 ± 0.14 pg/cell for Gd‐PAA12, and 0.81 ± 0.08, 0.95 ± 0.06, and 1.66 ± 0.08 pg/cell for Gd‐PASP11. The internalized Gd amounts of the OGMC Gd‐PAA12 and Gd‐PASP11 are higher than other reported GBCAs.^[^ ^21^ ^]^ The high cellular uptake demonstrates that MRI of cancer cells in vitro can be used to evaluate the MRI capability of Gd‐PAA12 or Gd‐PASP11.

*T*_1_‐weighted MR images (Figure 5D,E) (slice orientation: sagittal) reveal that the longer incubation time of Gd‐PAA12 or Gd‐PASP11, the stronger MR signal intensity for MCF‐7 cells within 2.0 h, which is consistent with the above‐mentioned results measured by ICP‐MS (Figure 5A–C). Figure 5F, G shows the *T*_1_‐weighted MR images (slice orientation: axial) and the corresponding ΔSNR of MCF‐7 cells incubated with Gd‐PAA12, Gd‐PASP11 or Gadavist, using DMEM as a control. Cells treated with Gd‐PAA12, Gd‐PASP11, or Gadavist all display obvious and similar MRI signal enhancement. The ΔSNR quantitative analyses also indicate that the signal enhancement of Gd‐PAA12 or Gd‐PASP11 is comparable to Gadavist. Because the cellular uptake of small molecule Gadavist (M_w_ = 604.7) via diffusion through cell membranes is much faster than that of the macrochelate Gd‐PAA12 or Gd‐PASP11 via phagocytosis,^[^ ^22^ ^]^ the similar MRI signal enhancement demonstrates the much stronger MRI capability of Gd‐PAA12 or Gd‐PASP11 than the commercial Gadavist.

The cytotoxicity of Gd‐PAA12 and Gd‐PASP11 were assessed on MCF‐7, 4T1, and U87 MG cells by methyl thiazolyl tetrazolium (MTT) assay, and compared with the commercial Gadavist (Figure S10, Supporting Information). The Gd‐PAA12 and Gd‐PASP11 show comparable cell viabilities to Gadavist on 4T1 or U87 MG cells at any Gd concentrations, and on MCF‐7 cells at low concentrations (*C*_Gd_ ≤ 125 µg mL^−1^). However, at high concentrations (*C*_Gd_ ≥ 250 µg mL^−1^), the cytotoxicity of Gd‐PAA12 or Gd‐PASP11 is notably lower than Gadavist on MCF‐7 cells (*P < 0.05, **P < 0.01). The lower cytotoxicity of the OGMC Gd‐PAA and Gd‐PASP compared with the commercial Gadavist can be ascribed to that Gd^3+^ is completely chelated with PAA or PASP forming 9 coordination bonds (i.e., the carboxyl groups of Gd‐PAA or the carboxyl and amino groups of Gd‐PASP are in excess to coordinate with Gd^3+^ as mentioned above) (Figure 1A,B). However, there are only 8 coordination bonds between Gd^3+^ and DO3A for the commercial Gadavist, and the remaining one interacts with H_2_O (Figure 1C), which is possible to interact with biological macromolecules in vivo, resulting in toxicity.^[^ ^23^ ^]^

### 2.3 Evaluation of Effectiveness and Safety on Mice

The hemocompatibility was further used to evaluate the biocompatibility of the OGMC Gd‐PAA12 and Gd‐PASP11 (Figure 5H,I). Pure water and PBS (pH = 7.4) were used as positive and negative controls, respectively. It is clear that the positive control of pure water has severe hemolysis of RBCs, while the hemolysis rate treated with PBS, Gd‐PAA12, and Gd‐PASP11 are all less than 2.0%. Thus, the developed Gd‐PAA12 and Gd‐PASP11 have good hemocompatibility.

Pharmacokinetic studies (Figure S11, Supporting Information) reveal that the blood circulation half‐lives of Gd‐PAA12 and Gd‐PASP11 are 16.67 min and 31.44 min, respectively, which are much shorter than the reported GONs because of the smaller sizes,^[^ ^7^, ^11^, ^17^ ^]^ and slightly longer than the commercial Gadavist (14.05 min) due to the larger M_w_ of the macrochelates. Clinically, the best time window for MRI (10–15 min) is near to the half‐life of commercial CAs (i.e., small molecule of Gd‐chelates), which is a little bit tight for MRI after Gd‐chelates administration. The slightly longer half‐lives of our Gd‐PAA12 and Gd‐PASP11 (16–32 min) overcome the problems of commercial GBCAs and reported GONs (usually too long a half‐life of several hours requiring a waiting period after drug administration).

The Gd contents in major organs and tumors of mice post‐injection of Gd‐PAA12 or Gd‐PASP11 were measured by ICP‐OES (Figure S12A,B, Supporting Information), indicating that both Gd‐PAA12 and Gd‐PASP11 can be excreted via the liver and kidneys due the medium sizes compared with commercial GBCAs and reported GONs. The urine and feces samples were subsequently studied within 24 h after *i.v*. injection of Gd‐PAA12 or Gd‐PASP11 (Figure S13, Supporting Information). The excreted of Gd content in urine with 0–8.0, 8.0–16, and 16–24 h were 46.18 ± 6.34, 9.53 ± 4.59, and 0.62 ± 0.43%ID/g for Gd‐PAA12, and 18.05 ± 1.48, 4.85 ± 0.70 and 1.17 ±  1.69%ID/g for Gd‐PASP11. The excreted Gd content in feces is much lower than that in urine. These results demonstrate that our OGMC Gd‐PAA12 and Gd‐PASP11 are mainly cleared through the renal routes and less from the liver.

Biosafety in vivo is of great significance for further biomedical applications. In vivo toxicity of mice was evaluated by blood routine and blood biochemical analyses at days 1.0, 7.0, and 21 post‐injection of Gd‐PAA12 or Gd‐PASP11. At the animal level, no visible differences in blood routine indicators (i.e., RBC, HGB, HCT, MCV, MCH, MCHC, WBC, and PLT) were observed on the Gd‐PAA12 or Gd‐PASP11 administered mice (Figures S14A and S15A). Assessment of hepatic function (i.e., AST, ALP, ALT, ALB) and renal function (i.e., BUN, TP, CREA) (Figures S14B and S15B) also confirm that the OGMC Gd‐PAA12 or Gd‐PASP11 do not cause obvious hepatotoxicity or nephrotoxicity. Besides, histological analyses of main organs (H&E staining) were performed with high doses of Gd‐PAA12 or Gd‐PASP11 (*C*_Gd_ = 10.0 mg kg^−1^) injected into healthy mice 2.0 or 30 days after administration (Figure S16, Supporting Information). As a result, the images of the heart, liver, spleen, lung, and kidneys in mice show no significant histological abnormalities, indicating that our OGMC Gd‐PAA12 or Gd‐PASP11 do not cause acute and long‐term toxicity. To study the deposition of gadolinium in the brain, brain tissues of mice at 24 or 72 h post‐injection of Gd‐PAA12 or Gd‐PASP11 were digested and quantified by ICP‐OES. As shown in Figure S17 (Supporting Information), the gadolinium contents at both 24 and 72 h are almost zero for both Gd‐PAA12 and Gd‐PASP11, indicating the low risks of Gd deposition in the brain.

**Figure 6:**
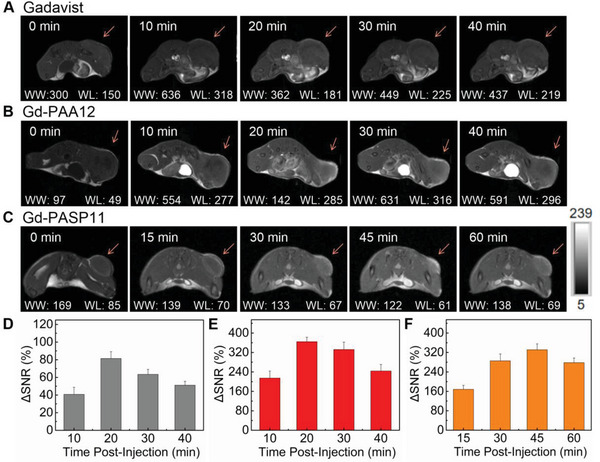
In vivo study of *T*_1_‐weighted MR imaging. A–C) In vivo *T*_1_‐weighted MR images of 4T1 tumor‐bearing mice on a 7.0 T MRI scanner (slice orientation: axial) after intravenous injection of Gadavist A), Gd‐PAA12 B), or Gd‐PASP11C). *C*_Gd_ = 5.0 mg kg^−1^. The MR images pre‐injection are identified as 0 min. WW and WL are the window width and window level values, respectively. D–F) Quantitative analysis of signal changes in tumors post‐injection of Gadavist D), Gd‐PAA12 E), or Gd‐PASP11 F). The color bar is the same for A–C).

The *T*_1_‐weighted MR images (slice orientation: axial) of 4T1 tumor‐bearing mice at predetermined times after *i.v*. injection of Gadavist, Gd‐PAA12, or Gd‐PASP11 (Gd dosage = 5.0 mg kg^−1^) were observed on a 7.0 T MRI scanner. Before drug administration, the tumor‐bearing mice were scanned and the images are labeled as 0 min. The tumors in the MR images are brightest at 20 min post‐injection of Gadavist (**Figure** **6**A), and then the brightness decreases gradually. However, the tumor brightness in the MR images increases rapidly post‐injection of Gd‐PAA12 or Gd‐PASP11 reaching the peak at 20 or 45 min without obvious decrease until 30 or 60 min, respectively (Figure 6B,C). That's because the blood circulation half‐lives of Gd‐PAA12 and Gd‐PASP11 (i.e., 16.67 and 31.44 min) are longer than that of the Gadavist (i.e., 14.05 min) due to their larger M_w_ (i.e., 3927 and 7272) compared with that of the Gadavist (604.71).

Quantitative analysis of signal changes in tumors post‐injection of Gadavist, Gd‐PAA12, or Gd‐PASP11 indicates that the maximum ΔSNR of tumor is only 82 ± 8.0% at 20 min post‐injection of Gadavist (Figure 6D), which are 364 ± 18% at 20 and 45 min post‐injection of Gd‐PAA12 (Figure 6E), and 331 ± 24% at 45 min post‐injection of Gd‐PASP11 (Figure 6F). The satisfactory in vivo MRI results are mainly attributed to the good blood compatibility and outstanding relaxivities of Gd‐PAA12 and Gd‐PASP11. Thus, it can be concluded that our OGMC Gd‐PAA12 and Gd‐PASP11 are more effective for *T*_1_‐weighted MRI than small‐molecule GBCAs. The MRI signal of the bladder in 4T1 tumor‐bearing mice gradually increased over time post‐injection of Gd‐PAA12 (Figure S18, Supporting Information). ΔSNR quantification results (261 ± 8.0% at 40 min) further confirm that it is mainly excreted through the kidneys. All of these in vivo experimental results suggest that the highly biocompatible macrochelates of Gd‐PAA12 and Gd‐PASP11 can be used as qualified *T*_1_‐weighted MRI CAs.

The possibility of industrial manufacture was further investigated for our Gd‐PAA12 and Gd‐PASP11. The synthesis conditions were optimized in 20 mL, 2.0 L, and 20 L reactors step by step. After freeze‐drying, the yields of Gd‐PAA12 and Gd‐PASP11 synthesized in 2.0 and 20 L of reactors are all around than 90%, and the production of Gd‐PAA12 and Gd‐PASP11 reaches hundred‐gram‐scale (Figure 1D–G; Figure S19, Supporting Information). The large‐scale synthesized Gd‐PAA12 and Gd‐PASP11 were tested on the 3.0 T and 7.0 T MRI scanners, and the relaxation rate plots are shown in Figures S20 and S21 (Supporting Information), with relaxivities summarized in **Table** **1**. It can be found that Gd‐PAA12 and Gd‐PASP11 still possess similarly outstanding relaxation properties (*r*_1_ > 50 mm ^−1^ s^−1^, *r*_2_/*r*_1_ < 1.6, 3.0 T) after step‐by‐step scaled‐up synthesis, suggesting the great potential of the weakly ferromagnetic OGMCs to be used as CAs for *T*_1_‐weighted MRI. The imaging principles of our Gd‐PAA12 and Gd‐PASP11 are the same as with the commercial Gd‐based MRI CAs. Although we focus on the tumor MRI in this study, Gd‐PAA12 and Gd‐PASP11 can also be used for MRI of other tissues and diseases.

**Table 1:** Relaxivities of Gd‐PAA12 and Gd‐PASP11 synthesized in reactors with different volumes

Sample Nomenclature	V [L]^a)^	H_0_ [T]^b)^	*r*_1_ [mm ^−1^ s^−1^]	*r*_2_ [mm ^−1^ s^−1^]	*r*_2_ / *r*_1_
Gd‐PAA12	0.02	3.0	56.23 ± 1.69	86.58 ± 1.18	1.54 ± 0.03
		7.0	17.18	92.55	5.39
	2.0	3.0	54.00	85.41	1.58
		7.0	16.95	93.66	5.53
	20	3.0	53.74	85.89	1.60
		7.0	16.84	91.97	5.46
Gd‐PASP11	0.02	3.0	54.00 ± 0.47	80.23 ± 0.80	1.49 ± 0.02
		7.0	19.60	76.84	3.92
	2.0	3.0	51.22	70.54	1.38
		7.0	16.80	67.81	4.04
	20	3.0	50.46	71.46	1.42
		7.0	16.81	70.81	4.21

^a)^ The volume of the used reactors

^b)^ The *r*_1_ and *r*_2_ values were measured on a MRI scanner system (7.0 T, Bruker, PharmaScan70/16 US), or a clinical MRI scanner system (Philips, Ingenia 3.0 T). Mean ± SD, *n* = 3.

## 3 Conclusions

In summary, to overcome the problems of commercial and reported GBCAs, we propose a new concept of OGMCs, which are constructed from the coordination between Gd^3+^ and macromolecules. To verify the practicality of the OGMC concept, in this study, some macromolecules were screened for Gd^3+^ coordination and two candidates (i.e., PAA and PASP) succeeded in OGMC formation, which was characterized by ITC, FT‐IR, ICP‐OES, ICP‐MS, DLS, GPC, PPMS, MRI scanner (3.0 and 7.0 T). Under optimized synthesis conditions, both Gd‐PAA12 and Gd‐PASP11 are outstanding *T*_1_‐weighted CAs owing to their super high *r*_1_ values (> 50 mm ^−1^ s^−1^, 3.0 T) and ultralow *r*_2_/*r*_1_ ratios (< 1.6, 3.0 T). The ferromagnetism of our OGMC Gd‐PAA12 and Gd‐PASP11 is completely different from the paramagnetism of the commercial and reported GBCAs, which demonstrates that our weakly ferromagnetic OGMCs are brand‐new GBCAs. The very weak ferromagnetism like our OGMCs (M_s_ < 1.0 emu g^−1^) is rarely reported and leads to a low *r*_2_, which is good for *T*_1_ MRI. Gd^3+^ almost cannot be released from the OGMC Gd‐PAA12 and Gd‐PASP11 because the carboxyl groups of Gd‐PAA12 and the carboxyl and amino groups of Gd‐PASP11 are excess to coordinate with Gd^3+^, and the coordination is firm, which greatly reduce Gd^3+^ release to ensure the biosafety for in vivo applications. The safety and *T*_1_‐weighted MRI efficacy of the OGMC Gd‐PAA12 and Gd‐PASP11 were further confirmed in cells and mice. More importantly, the synthesis method of the OGMCs is facile and easy to be scaled up. Therefore, the OGMC Gd‐PAA12 and Gd‐PASP11 can be used as superior *T*_1_‐weighted MRI CAs with promising translational possibility to replace the commercial Gd chelates.

## Conflict of Interest

The authors declare no conflict of interest.

## Supporting information

Supporting Information

## Data Availability

The data that support the findings of this study are available in the supplementary material of this article.
