# Supplementary material for: Facile Synthesis of Weakly Ferromagnetic Organogadolinium Macrochelates‐Based T1‐Weighted Magnetic Resonance Imaging Contrast Agents
Source: Adv Sci (Weinh). 2022 Nov 15;10(1):2205109. doi: 10.1002/advs.202205109 (PMC9811448; doi:10.1002/advs.202205109)
Supplement: Supplementary file 1 — Supporting Information [file ADVS-10-2205109-s001.pdf]

ADVANCED SCIENCE
Open Access

# Supporting Information

for *Adv. Sci.*, DOI 10.1002/advs.202205109

Facile Synthesis of Weakly Ferromagnetic Organogadolinium Macrochelates-Based $T_1$-Weighted Magnetic Resonance Imaging Contrast Agents

*Yudie Lu, Zhiyu Liang, Jie Feng, Lin Huang, Shuai Guo, Peiwei Yi, Wei Xiong, Sijin Chen, Sugeun Yang, Yikai Xu, Yan Li, Xiaoyuan Chen and Zheyu Shen**

# Supporting Information

**Facile Synthesis of Weakly Ferromagnetic Organogadolinium Macrochelates-Based $T_1$-Weighted Magnetic Resonance Imaging Contrast Agents**

*Yudie Lu, Zhiyu Liang, Jie Feng, Lin Huang, Shuai Guo, Peiwei Yi, Wei Xiong, Sijin Chen, Sugeun Yang, Yikai Xu, Yan Li, Xiaoyuan Chen, Zheyu Shen**

## Experimental section

*Materials*: Poly(acrylic acid sodium salt) (PAANa, average $M_w$ ~ 2100), poly(acrylic acid sodium salt) (average $M_w$ ~ 5100) and poly(acrylic acid, sodium salt) (solution, average $M_w$ ~ 8000, 45 wt % in $H_2O$) were purchased from Sigma-aldrich (USA). Gadolinium (III) nitrate hexahydrate ($Gd(NO_3)_3{\cdot}6H_2O$, 99.9 %), sodium of poly(aspartic acid) (PASPNa, $M_w$ ~ 7500), sodium of polyaspartic acid ($M_w$ ~ 1000), Rhodamine 6G (R6G) and sodium hydroxide (NaOH, 97 %) were purchased from Macklin (Shanghai, China). 2-(4-Amidinophenyl)-6-indolecarbamidine dihydrochloride (DAPI) and FITC-Phalloidin was purchased from Beyotime Biotechnology (Shanghai, China).

*Study design*: The main objective of this study was to develop novel OGMCs with excellent MRI performance as $T_1$-weighted MRI CAs. A variety of synthesis conditions were optimized and the OGMCs were extensively characterized. The kinetic stability and blood compatibility were verified by *in vitro* release experiments and hemolysis tests. The good biocompatibility was demonstrated by cytotoxicity tests, routine blood tests, mouse biochemical index analysis, and H & E staining analysis of major organs. The efficacy was assessed by the relaxation properties ($r_1$ value and $r_2/r_1$ ratio), MR imaging of *in vitro* tumor cells and

subcutaneous tumor-bearing mice. A facile synthesis method of the OGMCs was scaled up to hundred-gram-scale.

*Synthesis of weakly ferromagnetic organogadolinium macrochelates (OGMCs)*: PAANa ($M_w$ ~ 2100, 5100, or 8000) solution (4.0 mg/mL, 20 mL) was first purged with nitrogen (≥ 40 min) to remove oxygen. The polymer solution was kept at room temperature (25 $^{o}$C), or heated to reflux (100 $^{o}$C). After that, $Gd(NO_3)_3$ (30.0-250 mM, 0.40 mL) solution was added into the reaction system. The reaction was continued for 90 min under magnetic stirring at room temperature or 100 $^{o}$C to obtain the OGMC Gd-PAA. Finally, the solutions were cooled down to room temperature. The obtained OGMC Gd-PAA were purified by membrane dialysis ($M_w$ cut-off 6-8 kDa) against Milli-Q water for three days with water change twice a day. The purified OGMC Gd-PAA were concentrated *via* rotary evaporation.

The OGMC Gd-PASP was synthesized *via* the similar procedure with detailed conditions provided in the following tables.

Large-scale facile synthesis of the OGMC Gd-PAA or Gd-PASP was synthesized in a 2.0 or 20 L of reactor *via* the above-mentioned procedures with optimized conditions, in which the volumes of reactants were enlarged by same multiples.

*General measurement*: Gd concentrations ($C_{Gd}$) of the solutions were measured using an inductively coupled plasma optical emission spectrometer (ICP-OES, iCAP PRO Series, Thermo Fisher Scientific), or inductively coupled plasma mass spectrometry (ICP-MS, PerkinElmer Nexion 300). The structure of the OGMC Gd-PAA or Gd-PASP was verified by fourier transform infrared spectrometer (FT-IR, Nicolet IS50, Thermo Fisher Scientific). The thermodynamic parameters of the interaction between $Gd^{3+}$ and PAA (or PASP) were measured using an isothermal titration calorimetry (ITC, NANO ITC, TA instruments). The ITC experiments were conducted by placing PAANa (0.10 mM) in the reaction cell and

$Gd(NO_3)_3$ (10 mM) in the syringe, the titration volume was set at 10 μL, the titration interval was set at 200 s, and the titration times were 25 times. The molecular weight ($M_w$) and polydispersity (PDI) of PAA, Gd-PAA12, PASP and Gd-PASP11 were measured on a gel permeation chromatograph (GPC, 1260 Infinity II, Aglient) system, which was calibrated with polyethylene glycol (PEG). Pure water was used as mobile phase at a flow rate of 1.0 mL/min. Field-dependent magnetization curves of the OGMC Gd-PAA or Gd-PASP were measured by physical property measurement system (PPMS) at 298 K.

*MR imaging and relaxivity measurement*: The aqueous solutions of OGMC Gd-PAA or Gd-PASP were prepared with gradient $C_{Gd}$, and then phantom images and relaxation times were acquired on a 7.0 T MRI scanner (Bruker, PharmaScan70/16 US) or a clinical 3.0 T MRI scanner system (3.0 T, Philips, Ingenia, NL). The $r_1$ or $r_2$ relaxivities were calculated from the slope of the linear fitting lines of $1/T_1$ or $1/T_2$ versus Gd concentrations ($C_{Gd}$). For a 7.0 T MRI scanner, the parameters for $T_1$ measurements were set as follows: echo time (TE) = 7.3 ms, repetition time (TR) = 120 ms; the parameters for $T_2$ measurements were set as follows: TE= 120 ms, TR = 5000 ms. For a 3.0 T MRI scanner, the $T_1$ measurements sequence was: TE= 8.2 ms, TR = 200 ms; the parameters for $T_2$ measurements were set as follows: TE= 80 ms, TR = 5000 ms. Signal intensities were measured with Image J software. The signal-to-noise ratio (SNR) and ΔSNR were calculated using the formula (1) and (2).

$$SNR = SI_{mean} / SD_{noise} \quad (1)$$

$$\Delta SNR = (SNR_{sample} - SNR_{water}) / SNR_{water} \times 100\% \quad (2)$$

*Release behaviors of Gd from the OGMCs*: The OGMC Gd-PAA or Gd-PASP ($C_{Gd}$ = 1.0 mM, 10 mL) was respectively transferred into a dialysis bag, which was then placed in 500 mL PBS at pH 4.5 or 7.4. The PBS solutions with various pH values were stored at 37 $^{o}$C. At predetermined time intervals, 10 mL of the PBS solutions with different pH values were taken

and digested for ICP-MS analysis. The Gd release behavior at different pH values was monitored *via* a plot of the cumulative released Gd content (*i.e.*, the molar percentage of the released Gd to the total amount of Gd in OGMC Gd-PAA or Gd-PASP) as a function of incubation time.

*Hemolysis assay*: For hemolysis assay, red blood cells were first isolated by centrifugation of fresh blood from Balb/c mice (250 × g, 10 min). The concentration of the collected blood cells was then diluted to 2.0 % (v/v). The $C_{Gd}$ of OGMC Gd-PAA12 or Gd-PASP11 was respectively adjusted to 800, 400, 200, 100, 50 and 25 μM using PBS. After that, 500 μL of Gd-PAA12 or Gd-PASP11 solutions with various $C_{Gd}$ were respectively added into 500 μL of blood cells, and the mixtures were immediately incubated in 37 ± 0.5 $^{o}$C of water bath for 4.0 h. Under the same conditions, red blood cells were respectively mixed with pure water or PBS (pH = 7.4) as a positive or negative control. The samples were taken out and centrifuged (250 × g, 15 min) to remove intact red blood cells. 100 μL of the supernatants were added into a 96-well plate, and the absorbance was measured at 545 nm using a microplate reader (Synergy H1, BioTek Instruments). Finally, the percentage of hemolysis was determined as $(A_{sample} - A_0)/ (A_{100} - A_0) * 100\%$, where $A_{sample}$, $A_{100}$, and $A_0$ is the absorbance of the samples, the completely lysed red blood cells in pure water, and zero hemolysis in PBS. Three parallel groups were tested for each concentration.

*Cell culture*: Human breast cancer cell line MCF-7, murine breast cancer cell line 4T1, human glioblastoma cell line U87 MG cells, and human normal hepatocyte line LO2 were cultured in DMEM medium supplemented with 10 % fetal bovine serum (FBS, Gibco) and 1.0 % penicillin/streptomycin (Biosharp). The cells were incubated at 37 $^{o}$C in a humidified atmosphere containing 5.0 % of $CO_2$.

*MTT assay*: The cytotoxicity of OGMC Gd-PAA12, Gd-PASP11, and Gadavist (a commercial MRI contrast agent) were evaluated by MTT assay against MCF-7, 4T1, and U87 MG cells. Typically, cells in complete DMEM medium were seeded in 96-well plates at a density of 5.0 × $10^3$ cells per well. After overnight incubation, the growth medium was replaced with a fresh one (without FBS) containing various $C_{\mathrm{Gd}}$ of Gd-PAA12, Gd-PASP11, or Gadavist. After 2.0 h of incubation, the growth medium was replaced with complete medium. After further 24 h of incubation, 10 μL MTT solution (5.0 mg/mL in PBS) was added to each well of the 96-well plates. After an additional 4.0 h of incubation, the growth medium was removed and the resulted formazan crystals in each well were dissolved with 150 μL of dimethyl sulfoxide (DMSO), and the absorbance of each well was recorded at a wavelength of 490 nm using a microplate reader (Synergy H1, BioTek Instruments).

*Cellular uptake*: The cellular uptake of OGMC Gd-PAA12 or Gd-PASP11 was first qualitatively measured by a confocal laser scanning microscopy (CLSM, A1 HD25, Nikon). Typically, LO2, MCF-7 or 4T1 cells were treated with R6G-labeled Gd-PAA12 or Gd-PASP11 ($C_{\mathrm{Gd}}$ = 0.50 mM) in the culture medium (without FBS) at 37 $^{\mathrm{o}}$C for 2.0 h. After being washed three times with PBS, the cells were fixed with fresh 4.0 % of formaldehyde for 15 min, permeabilized with 0.10 % of Triton X-100 for 5.0 min, blocked with 1.0 % of BSA for 30 min, treated with FITC-Phalloidin (0.50 μg/mL) for 45 min, and finally treated with DAPI (5.0 μg/mL) for 5.0 min at room temperature. After that, the CLSM images of the samples were observed on a CLSM imaging system.

The cellular uptake of OGMC Gd-PAA12 or Gd-PASP11 was also quantitatively determined by ICP-MS. Briefly, 2.0 mL of MCF-7, 4T1 or U87 MG cells in complete DMEM medium were seeded in 6-well plates at a density of 2.0 × $10^5$ cells per well, and incubated at 37 $^{\mathrm{o}}$C for 24 h. The growth medium was then replaced with a fresh one (2.0 mL, without FBS) without or with Gd-PAA12, or Gd-PASP11 ($C_{Gd}$ = 0.10 mM). After incubation at different

times, the cells were washed twice with PBS, treated with trypsin for 3.0 min, and then centrifuged at 800 × g for 3.0 min. The obtained cells were finally digested for Gd measurement by ICP-MS.

*MRI studies on cells*: The OGMC Gd-PAA12 or Gd-PASP11 was used for MR imaging of cancer cells compared with Gadavist. Typically, 8.0 mL of MCF-7 cells in complete growth medium were seeded in culture dish with a cell density of $2.0 \times 10^5$ cells/mL and allowed to adhere at 37 $^oC$ for 24 h. The growth medium was then replaced with fresh one (without FBS) without or with Gd-PAA12, Gd-PASP11, or Gadavist ($C_{Gd}$ = 0.10 mM). After further 2.0 h of incubation, the cells were washed with PBS, trypsinized, centrifuged, and transferred into 0.2 mL of centrifuge tubes. The obtained cells were used for scanning on a Bruker MRI scanner (7.0 T, PharmaScan70/16 US, Bruker, US, TE = 6.3 ms, TR = 300 ms, slices thickness = 0.80 mm, FOV = 3.0 cm).

*Tumor model*: All animal procedures were performed in accordance with the guidelines for Care and Use of Laboratory Animals of Southern Medical University, and approved by the Animal Ethics Committee of Southern Medical University. The assigned approval/accreditation number is SCXK 2016-0041. Female Balb/c mice (6~8 weeks old, 18~22 g) were purchased from the Experimental Animal Center of Southern Medical University (Guangzhou, China). The 4T1 tumor-bearing nude mice were prepared by subcutaneously inoculating 4T1 cells ($2.0 \times 10^6$ cells in 100 μL PBS) into the right leg of each mouse.

*In vivo study of $T_1$-weighted MR imaging*: The 4T1 tumor-bearing mice were anaesthetized by isoflurane (1.0-2.0 %) in oxygen and placed in an animal-specific body coil for MRI data acquisition (7.0 T, PharmaScan70/16 US, Bruker). Mice were kept warm by circulating warm

water (37 °C), and were placed in a stretched prone position with a respiratory sensor during the experiments. $T_1$-weighted images were acquired pre- and post-injection (intravenously) of Gd-PAA12, Gd-PASP11 or Gadavist (Gd dosage = 5.0 mg/kg) by a multi-slice multi-echo sequence using the parameters as follows: repetition time (TR) = 300 ms, echo time (TE) = 6.1 ms, flip angle = 180$^{o}$, matrix size = 256 × 256, field of view (FOV) = 4.0 × 4.0 cm$^2$, slices = 16, slice thickness = 0.80 mm. Signal intensities were measured with the Image J software. The SNR and ΔSNR were calculated using the formula (3) and (4).

$$SNR = SI_{mean} / SD_{noise} \quad (3)$$

$$\Delta SNR = (SNR_{post} - SNR_{pre}) / SNR_{pre} \times 100\% \quad (4)$$

*In vivo pharmacokinetics and biodistribution of the OGMCs*: For pharmacokinetic studies, healthy female Balb/c mice (20 ± 2 g) were injected (*i.v.*) with Gd-PAA12, Gd-PASP11, or Gadavist *via* tail vein, respectively (Gd dosage = 5.0 mg/kg, $n$ = 3). The blood samples (around 20 μL) were drawn from retro-orbital sinus at predetermined times after injection, and then thoroughly digested using concentrated nitric acid. The liquid was evaporated under heating. The sample residues were dissolved in 2.0 % of diluted nitric acid, and the concentration of Gd ($C_{Gd}$) was detected by ICP-OES.

For biodistribution analysis, the 4T1 tumor-bearing mice were injected (*i.v.*) with Gd-PAA12 or Gd-PASP11 (Gd dosage = 5.0 mg/kg). The main organs (heart, liver, spleen, lung, kidney, muscle, brain) and tumors were excised at 30 min, 24 h, or 72 h post-injection, and then completely digested in heated concentrated nitric acid. The liquid was evaporated under heating. The sample residues were dissolved in 2.0 % of diluted nitric acid, and the $C_{Gd}$ was measured by ICP-OES. The biodistribution of Gd was calculated as a percentage of injected dose per gram of tissue (I.D.%/g).

*Metabolism of the OGMCs*: The 4T1 tumor-bearing mice were injected (*i.v.*) with Gd-PAA12 or Gd-PASP11 (Gd dosage = 5.0 mg/kg). The metabolites (*i.e.*, urine and feces) were collected every 8.0 h within 24 h after injection, and then completely digested in heated concentrated nitric acid. The liquid was evaporated under heating. The sample residues were dissolved in 2.0 % of diluted nitric acid, and the $C_{Gd}$ was measured by ICP-OES. The Gd content was calculated as a percentage of injected dose per gram of urine or feces (I.D.%/g) for metabolism analysis.

*Biosafety evaluation of the OGMCs*: The biosafety of OGMC Gd-PAA12 or Gd-PASP11 was investigated on healthy Balb/c mice by the blood routine analyses, blood biochemical analyses, and histological analyses of main organs.

Balb/c mice were randomly divided into control group and experimental group. Experimental mice were intravenously injected with Gd-PAA12 or Gd-PASP11 (Gd dosage = 5.0 mg/kg, 200 μL). Blood samples were harvested for blood routine analyses, or blood biochemical analyses at day 1.0, 7.0 or 21. The mice injected with PBS were used as blank control.

The major organs of the mice were excised and subjected to histological analyses at day 2.0 or 30 post-injection (*i.v.*) of PBS, Gd-PAA12 or Gd-PASP11 (Gd dosage = 10.0 mg/kg, 200 μL). Hematoxylin and eosin (H&E) staining was performed on major organs including the heart, liver, spleen, lung and kidney, which were observed by a microscope.

*Statistical analysis*: Statistical analysis of data was performed with Student's *t*-test or one-way analysis of variance (ANOVA). Data were presented as mean ± SD. The level of significance was defined as $^{*}p < 0.05$, $^{**}p < 0.01$, $^{***}p < 0.001$, or $^{****}p < 0.0001$.

**Table S1.** Characterization results of the OGMC Gd-PAA1-3 and Gd-PASP1, 2.

| Sample Nomenclature | $M_w$ [a] | Gd Recovery [%] [b] | $H_0$ [T] | $r_1$ [$mM^{-1}$ $s^{-1}$] [c] | $r_2$ [$mM^{-1}$ $s^{-1}$] [c] | $r_2$ / $r_1$ [c] |
|---|---|---|---|---|---|---|
| Gd-PAA1 | 2100 | 91.1 | 3.0 | 46.41±0.97 | 69.97±1.80 | 1.51±0.07 |
| Gd-PAA2 | 5100 | 94.9 | 3.0 | 49.80±0.88 | 80.94±3.21 | 1.62±0.08 |
| Gd-PAA3 | 8000 | 91.3 | 3.0 | 48.46±1.04 | 82.60±4.50 | 1.71±0.06 |
| Gd-PASP1 | 1000 | 87.0 | 3.0 | 37.19±1.71 | 53.38±2.32 | 1.44±0.06 |
| Gd-PASP2 | 7500 | 93.0 | 3.0 | 49.77±0.79 | 65.10±2.76 | 1.31±0.07 |

[a]Relative molecular weight ($M_w$) of the reactant PAA or PASP; [b]Calculated from the molar percentage of Gd in the obtained Gd-PAA or Gd-PASP to that in the feeding materials; [c]The $r_1$ and $r_2$ values were measured on a clinical MRI scanner system (3.0 T, Philips, Ingenia, NL). Mean ± SD, $n$ = 3.

**Table S2.** Synthesis conditions and characterization results of the OGMC Gd-PAA4-11.

| Sample Nomenclature | $C_{PAA}$ [mg/mL][a)] | $C_{Gd(NO3)3}$ [mM][a)] | pH | Polymer/ Gd Mass Ratio[b)] | Gd Recovery [%][c)] | $H_0$ [T] | $r_1$ [$mM^{-1}$ $s^{-1}$][d)] | $r_2$ [$mM^{-1}$ $s^{-1}$][d)] | $r_2$ / $r_1$[d)] |
|---|---|---|---|---|---|---|---|---|---|
| Gd-PAA4 | 4 | 125 | 7.0 | 10.17 | 90.3 | 3.0 | 48.73±1.71 | 67.84±2.20 | 1.39±0.07 |
| Gd-PAA5 | 4 | 125 | 8.0 | 10.17 | 94.6 | 3.0 | 50.25±0.26 | 79.20±0.94 | 1.58±0.02 |
| Gd-PAA6 | 4 | 125 | 9.0 | 10.17 | 95.6 | 3.0 | 50.77±0.72 | 78.09±1.75 | 1.54±0.02 |
| Gd-PAA7 | 4 | 125 | 10.0 | 10.17 | 91.6 | 3.0 | 54.34±2.24 | 82.61±2.43 | 1.52±0.06 |
| Gd-PAA8 | 4 | 125 | 11.0 | 10.17 | 95.0 | 3.0 | 50.55±1.31 | 79.65±3.05 | 1.58±0.03 |
| Gd-PAA9 | 4 | 30 | 10.0 | 42.40 | 98.7 | 3.0 | 52.56±1.05 | 85.19±2.89 | 1.62±0.09 |
| Gd-PAA10 | 4 | 62.5 | 10.0 | 20.35 | 97.5 | 3.0 | 55.01±0.55 | 83.75±0.96 | 1.52±0.03 |
| Gd-PAA11 | 4 | 250 | 10.0 | 5.09 | 92.3 | 3.0 | 41.17±2.35 | 64.08±0.17 | 1.56±0.09 |

[a)]Concentration of the feeding PAA ($M_w$ = 5100) and $Gd(NO_3)_3$ before reaction; [b)]Calculated from the mass ratio of polymers to Gd in the feeding materials; [c)]Calculated from the molar percentage of Gd in the obtained Gd-PAA to that in the feeding materials; [d)]The $r_1$ and $r_2$ values were measured on a clinical MRI scanner system (3.0 T, Philips, Ingenia, NL). Mean ± SD, $n$ = 3.

**Table S3.** Synthesis conditions and characterization results of the OGMC Gd-PASP3-10.

| Sample Nomenclature | $C_{PASP}$ [mg/mL][a)] | $C_{Gd(NO3)3}$ [mM][a)] | pH | Polymer/ Gd Mass Ratio[b)] | Gd Recovery [%][c)] | $H_0$ [T] | $r_1$ [$mM^{-1}$ $s^{-1}$][d)] | $r_2$ [$mM^{-1}$ $s^{-1}$][d)] | $r_2$ / $r_1$[d)] |
|---|---|---|---|---|---|---|---|---|---|
| Gd-PASP3 | 4 | 125 | 7.0 | 10.17 | 77.5 | 3.0 | 51.99±2.48 | 68.26±3.34 | 1.32±0.11 |
| Gd-PASP4 | 4 | 125 | 8.0 | 10.17 | 87.2 | 3.0 | 52.79±0.66 | 78.60±1.63 | 1.49±0.03 |
| Gd-PASP5 | 4 | 125 | 9.0 | 10.17 | 89.1 | 3.0 | 53.14±1.48 | 92.94±3.13 | 1.75±0.08 |
| Gd-PASP6 | 4 | 125 | 10.0 | 10.17 | 91.3 | 3.0 | 53.68±0.76 | 81.6±0.58 | 1.51±0.03 |
| Gd-PASP7 | 4 | 125 | 11.0 | 10.17 | 82.9 | 3.0 | 50.53±1.30 | 67.71±3.92 | 1.34±0.05 |
| Gd-PASP8 | 4 | 30 | 10.0 | 42.40 | 99.5 | 3.0 | 46.81±1.55 | 70.18±1.91 | 1.50±0.09 |
| Gd-PASP9 | 4 | 62.5 | 10.0 | 20.35 | 89.2 | 3.0 | 50.99±0.91 | 73.11±4.01 | 1.43±0.07 |
| Gd-PASP10 | 4 | 250 | 10.0 | 5.09 | 23.7 | 3.0 | 52.02±1.69 | 74.36±0.30 | 1.43±0.04 |

[a)]Concentration of the feeding PASP ($M_w$ = 7500) and $Gd(NO_3)_3$ before reaction; [b)]Calculated from the mass ratio of polymers to Gd in the feeding materials; [c)]Calculated from the molar percentage of Gd in the obtained Gd-PASP to that in the feeding materials; [d)]The $r_1$ and $r_2$ values were measured on a clinical MRI scanner system (3.0 T, Philips, Ingenia, NL). Mean ± SD, $n$ = 3.

**Table S4.** Synthesis conditions and characterization results of the OGMC Gd-PAA12-15 and Gd-PASP11-14.

| Sample Nomenclature | $T$ [°C][a)] | Nitrogen[a)] | Gd Recovery [%][b)] | $H_0$ [T] | $r_1$ [$mM^{-1}$ $s^{-1}$][c)] | $r_2$ [$mM^{-1}$ $s^{-1}$][c)] | $r_2$ / $r_1$[c)] |
|---|---|---|---|---|---|---|---|
| Gd-PAA12 | 100 | Yes | 95.5 | 3.0 | 56.23±1.69 | 86.58±1.18 | 1.54±0.03 |
| | | | | 7.0 | 17.18 | 92.55 | 5.39 |
| Gd-PAA13 | 100 | No | 97.9 | 3.0 | 51.44±2.32 | 82.04±1.85 | 1.60±0.04 |
| | | | | 7.0 | 16.65 | 82.71 | 4.97 |
| Gd-PAA14 | 25 | Yes | 95.3 | 3.0 | 51.62±0.74 | 84.04±0.93 | 1.63±0.01 |
| | | | | 7.0 | 16.91 | 86.25 | 5.10 |
| Gd-PAA15 | 25 | No | 94.8 | 3.0 | 49.99±0.55 | 77.38±1.93 | 1.55±0.03 |
| | | | | 7.0 | 16.34 | 82.25 | 5.03 |
| Gadavist | - | - | - | 3.0 | 4.56±0.01 | 4.59±0.05 | 1.01±0.01 |
| | | | | 7.0 | 3.77 | 5.56 | 1.47 |
| Magnevist | - | - | - | 3.0 | 4.52±0.16 | 4.73±0.15 | 1.05±0.02 |
| | | | | 7.0 | 3.68 | 5.38 | 1.46 |
| $Gd(NO_3)_3$ | - | - | - | 3.0 | 10.99±0.73 | 11.78±0.51 | 1.07±0.03 |
| | | | | 7.0 | 8.92 | 12.90 | 1.45 |
| Gd-PASP11 | 100 | Yes | 92.8 | 3.0 | 54.00±0.47 | 80.23±0.80 | 1.49±0.02 |
| | | | | 7.0 | 19.60 | 76.84 | 3.92 |
| Gd-PASP12 | 100 | No | 93.5 | 3.0 | 51.24±0.95 | 73.85±0.90 | 1.44±0.03 |
| | | | | 7.0 | 18.00 | 63.17 | 3.51 |
| Gd-PASP13 | 25 | Yes | 90.9 | 3.0 | 49.88±1.47 | 65.02±2.19 | 1.3±0.01 |
| | | | | 7.0 | 17.44 | 69.62 | 3.99 |
| Gd-PASP14 | 25 | No | 89.7 | 3.0 | 50.34±0.81 | 74.96±0.57 | 1.49±0.01 |
| | | | | 7.0 | 17.44 | 72.00 | 4.13 |

[a)]The reactions were protected under nitrogen atmosphere or not; [b)]Calculated from the molar percentage of Gd in the obtained Gd-PAA or Gd-PASP to that in the feeding materials; [c)]The $r_1$ and $r_2$ values were measured on a MRI scanner system (7.0 T, Bruker, PharmaScan70/16 US), or a clinical MRI scanner system (3.0 T, Philips, Ingenia, NL). Mean ± SD, $n$ = 3.

**Table S5.** Thermodynamic properties. Thermodynamic parameters of the interaction between $Gd^{3+}$ and PAA (or PASP) measured by ITC.

| Interaction | Kd [M] | $n$[a)] | ΔH [kcal/mol] | ΔS [cal/mol·K] |
|---|---|---|---|---|
| $Gd^{3+}$ and PAA | $2.26 \times 10^{-7}$ | 4.7 | 9.617 | 62.66 |
| $Gd^{3+}$ and PASP | $6.89 \times 10^{-7}$ | 5.7 | 6.883 | 51.28 |

[a)]The stoichiometric ratio of $Gd^{3+}$ to PAA or PASP.

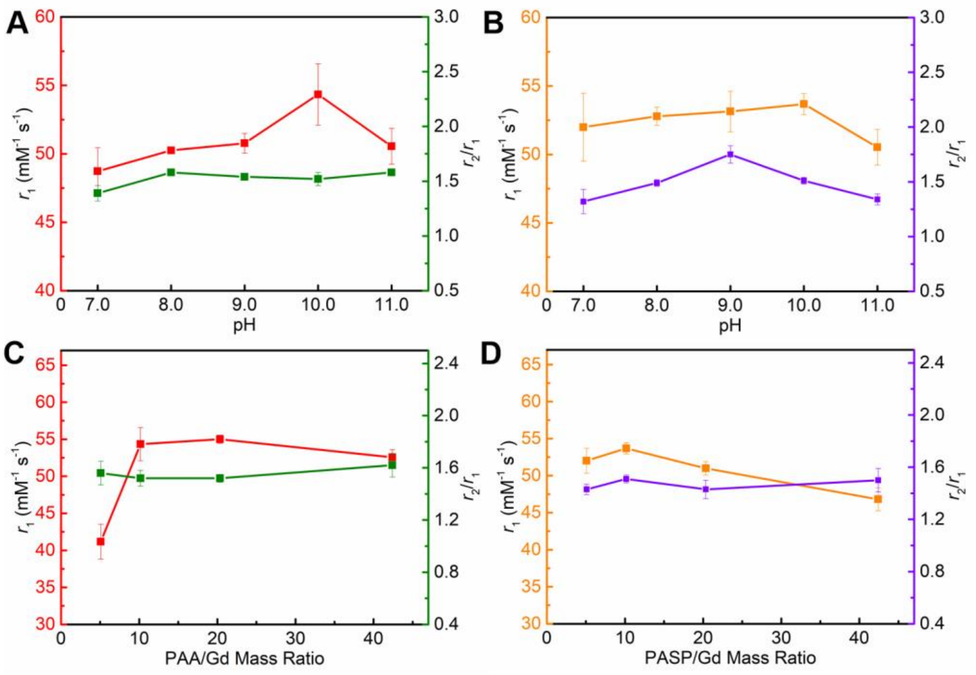


**Figure S1.** Influence of the pH value (A, B), or polymer/Gd molar ratios (C, D) on the $r_1$ value or $r_2/r_1$ ratio for Gd-PAA4-11 (A, C), or Gd-PASP3-10 (B, D). Mean ± SD, $n$ = 3.

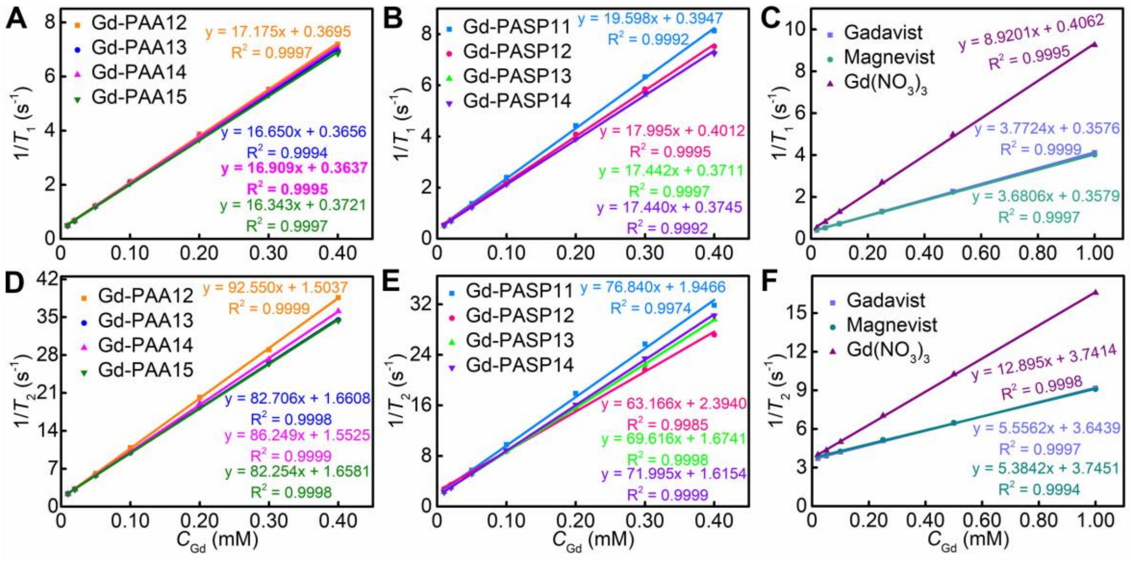


**Figure S2.** $T_1$ relaxation rate ($1/T_1$) (A-C) or $T_2$ relaxation rate ($1/T_2$) (D-F) plotted as a function of $C_{Gd}$ for Gd-PAA12-15 (A, D), and Gd-PASP11-14 (B, E) compared with Gadavist, Magnevist and $Gd(NO_3)_3$ (C, F) at 7.0 T.

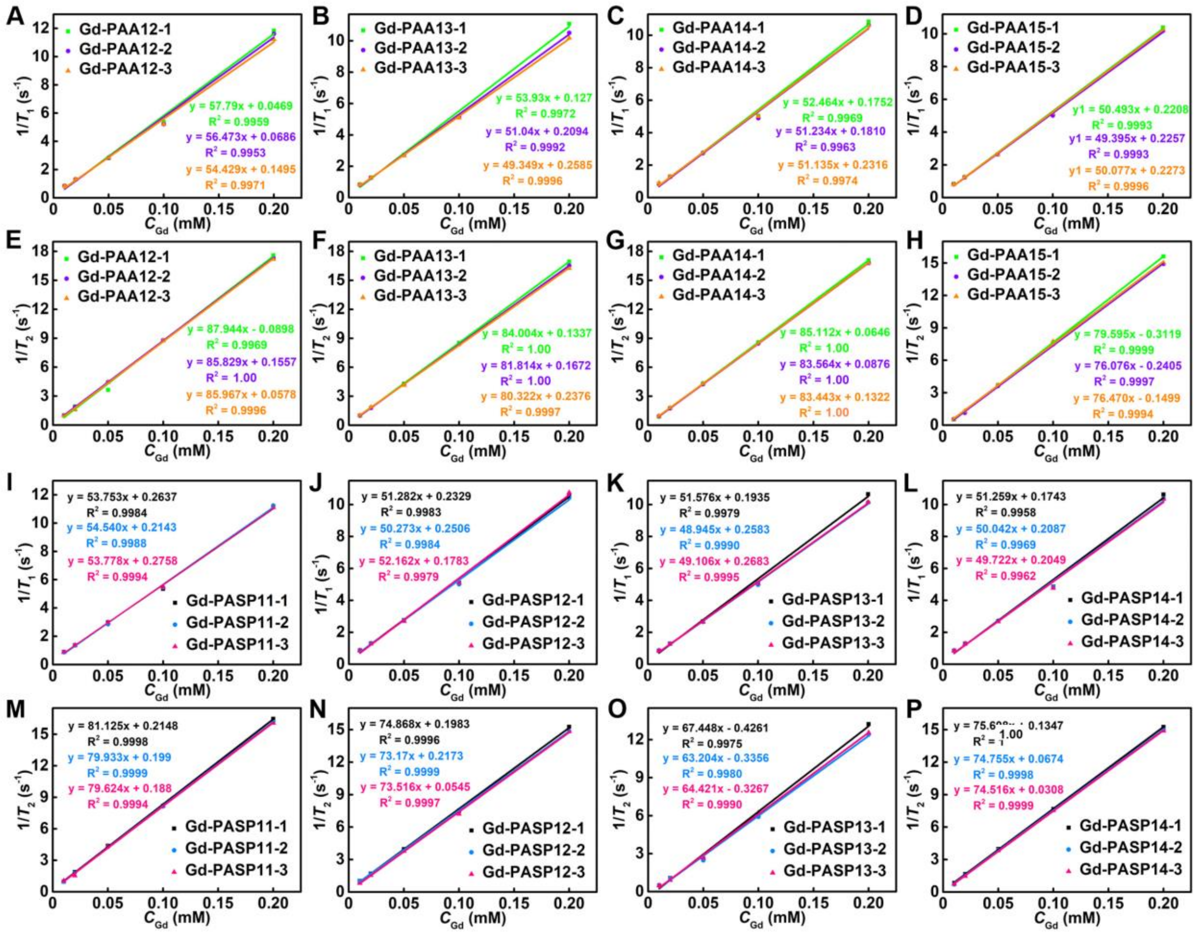

**Figure S3.** $T_1$ relaxation rate ($1/T_1$) (A-D, I-L) or $T_2$ relaxation rate ($1/T_2$) (E-H, M-P) plotted as a function of $C_{Gd}$ for Gd-PAA12-15 (A-H), or Gd-PASP11-14 (I-P) at 3.0 T ($n$ = 3).

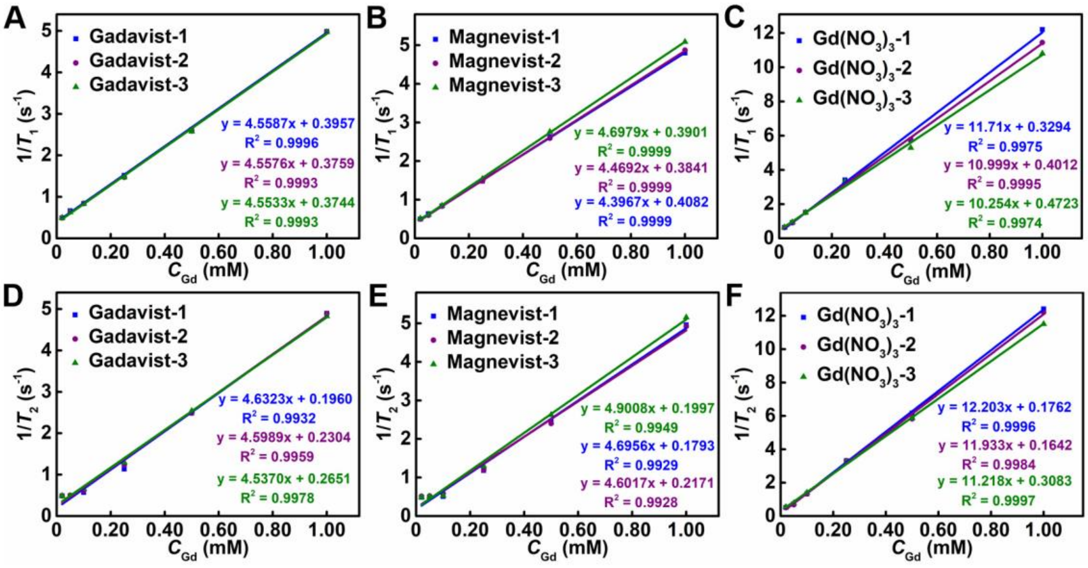


**Figure S4.** $T_1$ relaxation rate ($1/T_1$) (A-C) or $T_2$ relaxation rate ($1/T_2$) (D-F) plotted as a function of $C_{Gd}$ for Gadavist (A, D), Magnevist (B, E), or $Gd(NO_3)_3$ (C, F) at 3.0 T ($n$ = 3).

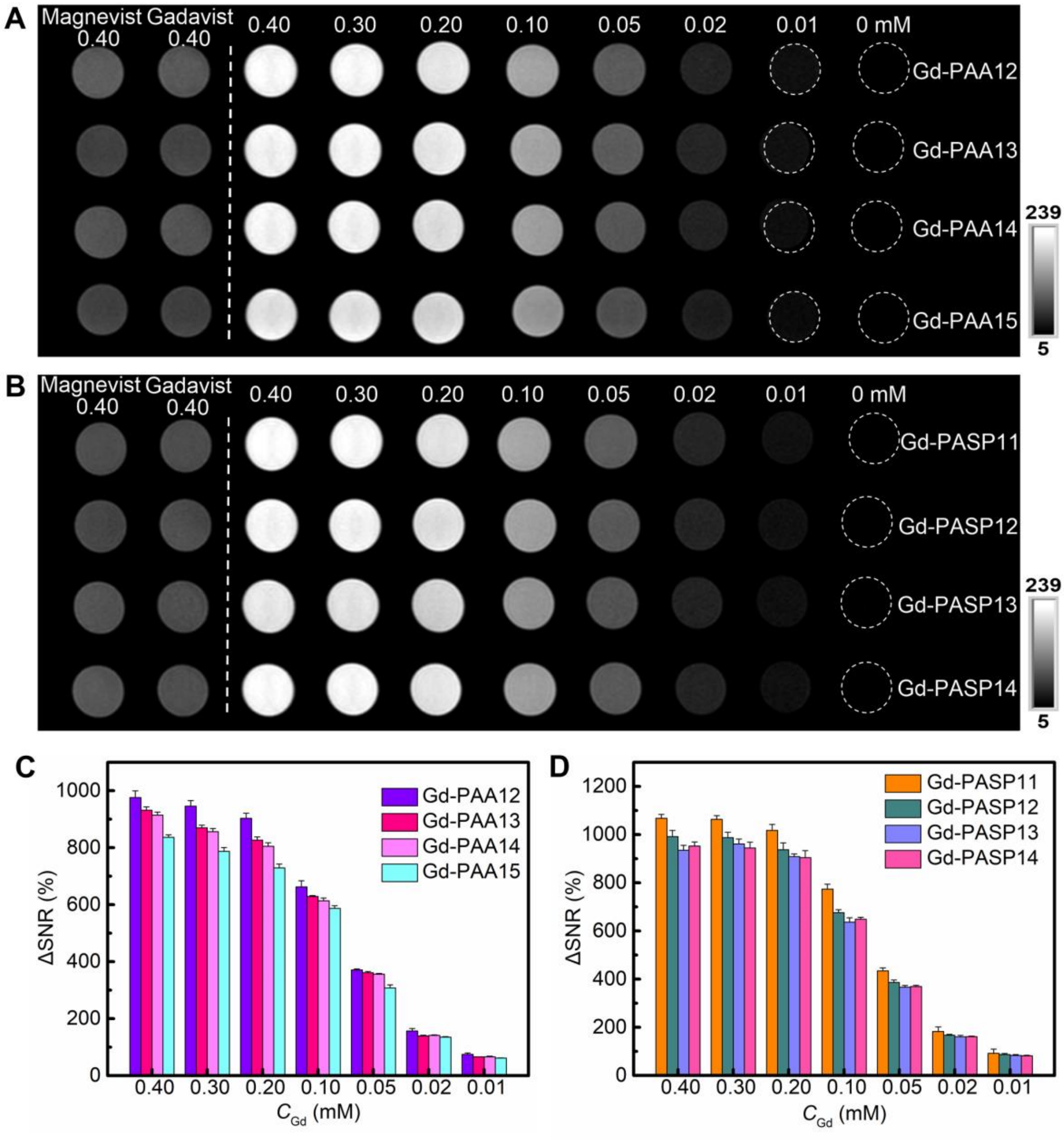


**Figure S5.** (A, B) $T_1$-weighted MR images of Gd-PAA12-15 (A), or Gd-PASP11-14 (B) with various $C_{Gd}$ (0 ~ 0.40 mM) compared with the commercial Magnevist and Gadavist observed by a 3.0 T clinical MRI system. (C, D) ΔSNR of the MR images for Gd-PAA12-15 (A), or Gd-PASP11-14 (B) with various $C_{Gd}$ ($n$ = 3).

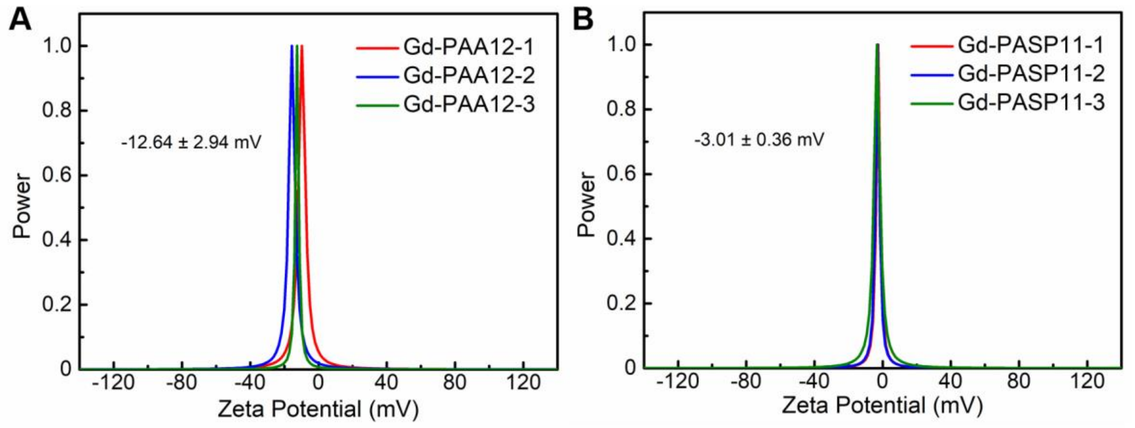


**Figure S6.** Zeta potential measurement for three different batches of Gd-PAA12 (A), or Gd-PASP11 (B).

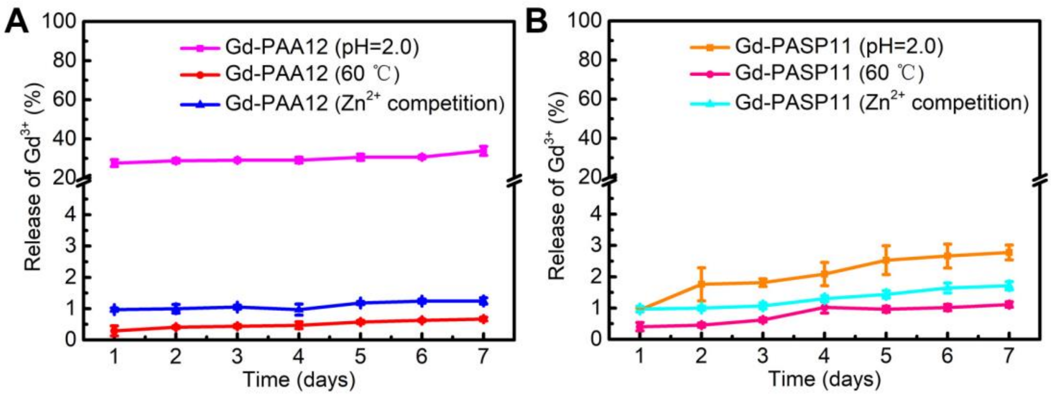


**Figure S7.** The time-dependent release of free $Gd^{3+}$ from Gd-PAA12 (A) or Gd-PASP11 (B) in PBS of pH 2.0 at 37°C, pH 7.4 at 60°C, or pH 7.4 at 37°C with double amount of $Zn^{2+}$ compared with Gd in Gd-PAA12 or Gd-PASP11.

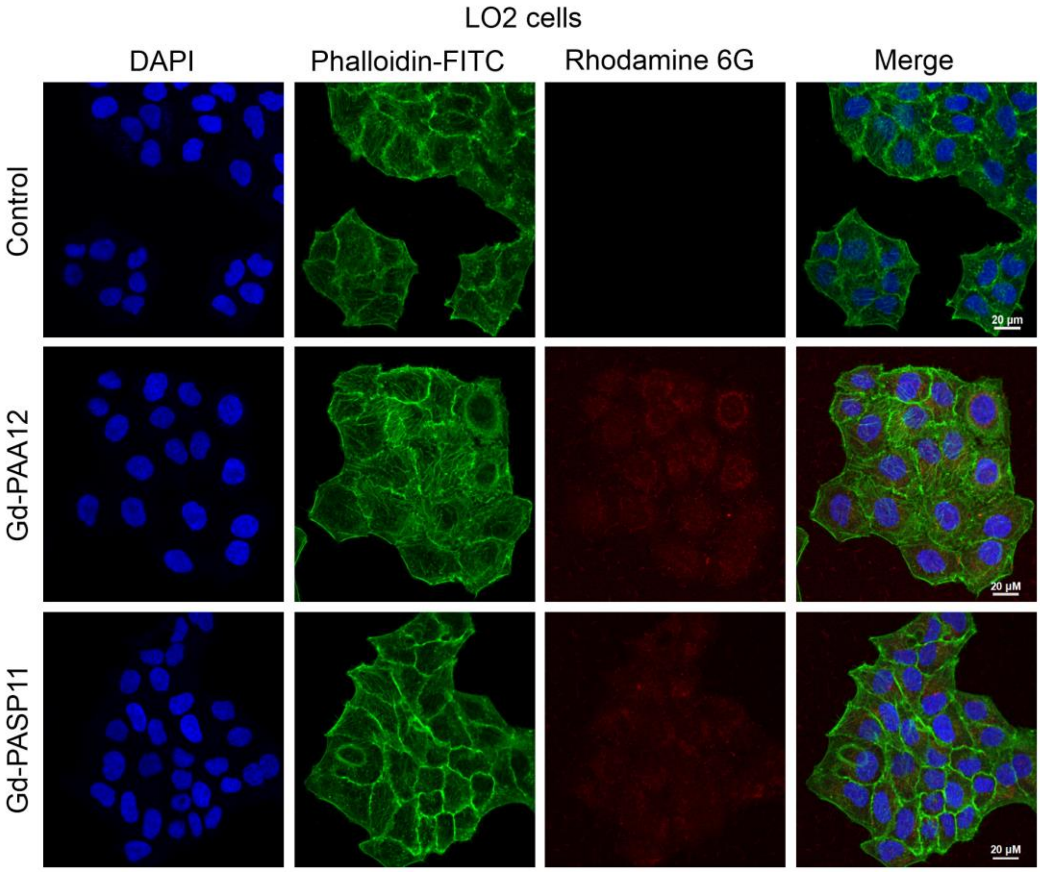


**Figure S8.** CLSM images of LO2 cells treated with R6G-Gd-PAA12, or R6G-Gd-PASP11 for 2.0 h at 37 $^{o}$C. The untreated cells were used as a control. The nucleus stained with DAPI is blue, and the cytoskeleton stained with phalloidin-FITC is green. The Rhodamine 6G-labeled OGMCs are red.

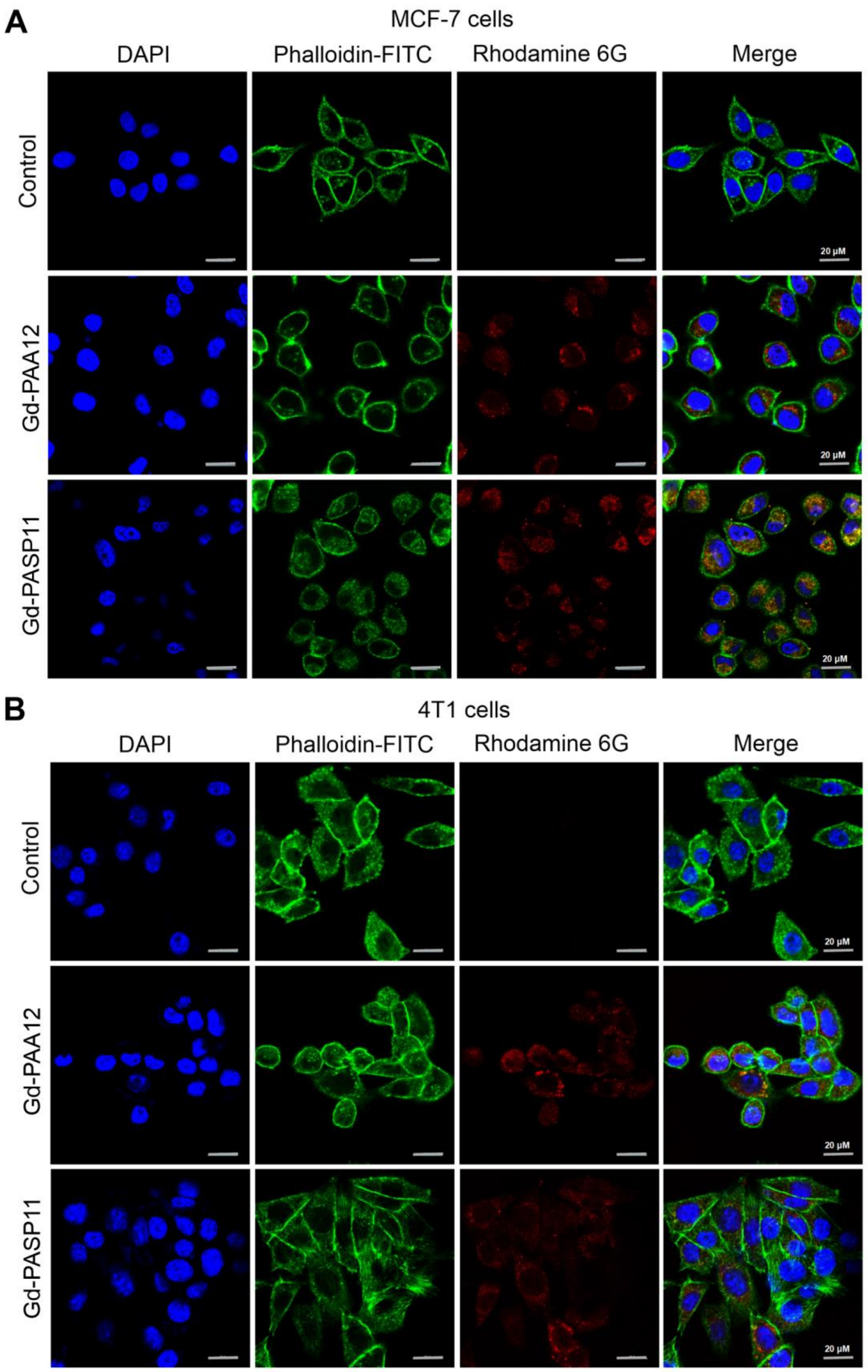


**Figure S9.** CLSM images of MCF-7 (A), or 4T1 (B) cells treated with R6G-Gd-PAA12, or R6G-Gd-PASP11 for 2.0 h at 37 $^{o}$C.

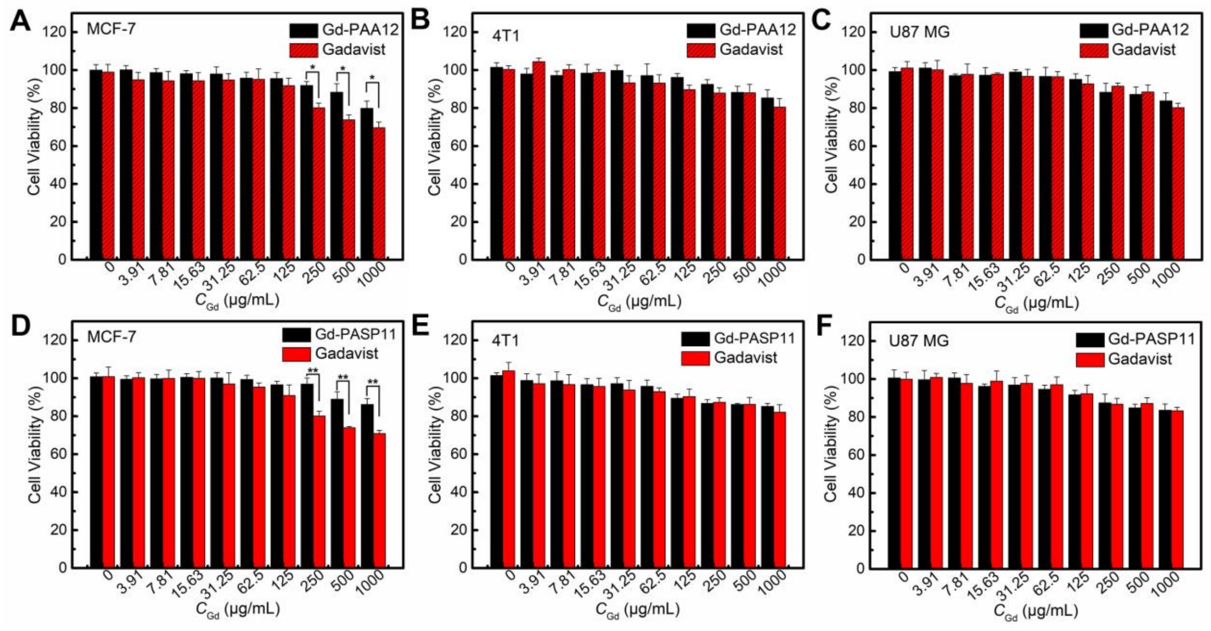


**Figure S10.** Viabilities of MCF-7 (A, D), 4T1 (B, E) and U87 MG cells (C, F) treated with Gd-PAA12 (A-C), or Gd-PASP11 (D-F) compared with Gadavist (control) in a Gd concentration range of 0-1000 μg/mL.

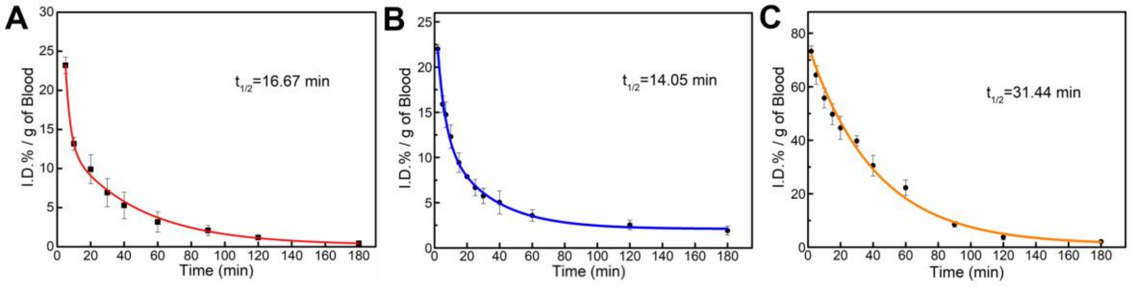


**Figure S11.** Blood clearance profiles of Gd-PAA12 (A), Gadavist (B), or Gd-PASP11 (C) in healthy Balb/c mice by tracking the Gd concentration in blood at different time intervals after *i.v.* injection ($n$ = 3). Gd dosage = 5.0 mg/kg.

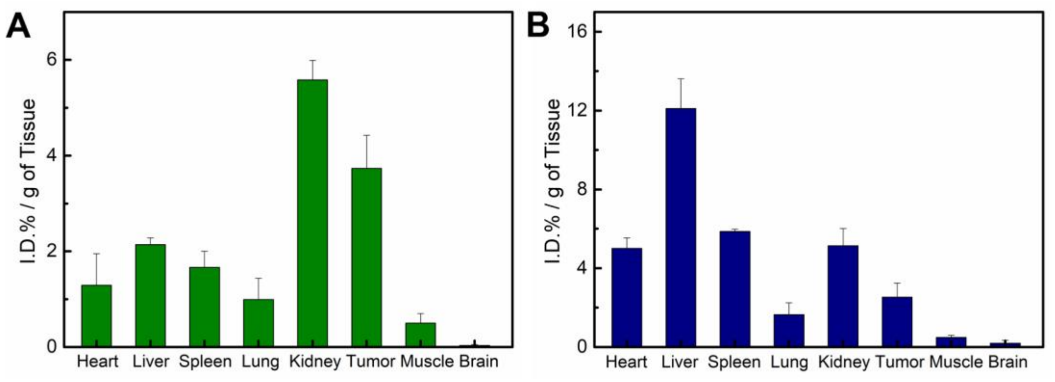


**Figure S12.** *In vivo* biodistribution of Gd level in 4T1 tumor-bearing mice at 30 min post-injection of Gd-PAA12 (A), or Gd-PASP11 (B) *via* tail vein ($n$ = 3). Gd dosage = 5.0 mg/kg.

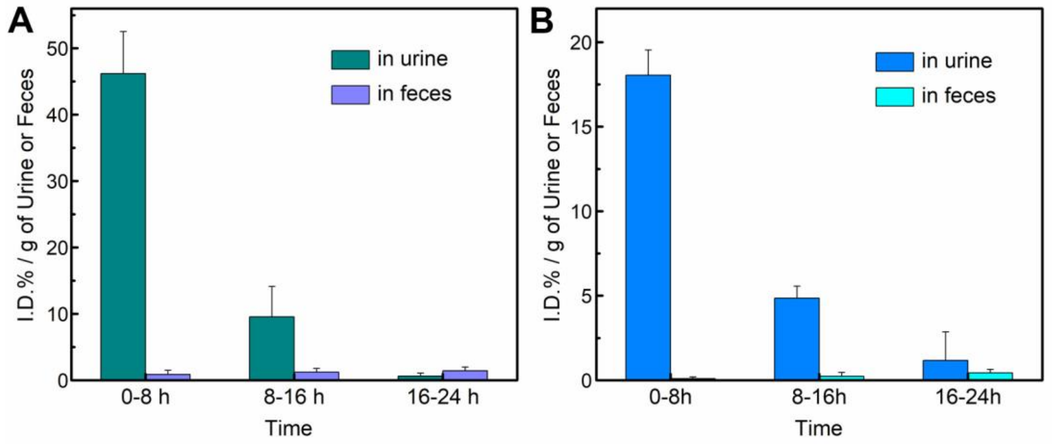


**Figure S13.** Excreted Gd content in urine or feces of healthy Balb/c mice within 24 h after *i.v.* injection of Gd-PAA12 (A), or Gd-PASP11 (B). Mean ± SD, $n$ = 5. Gd dosage = 5.0 mg/kg.

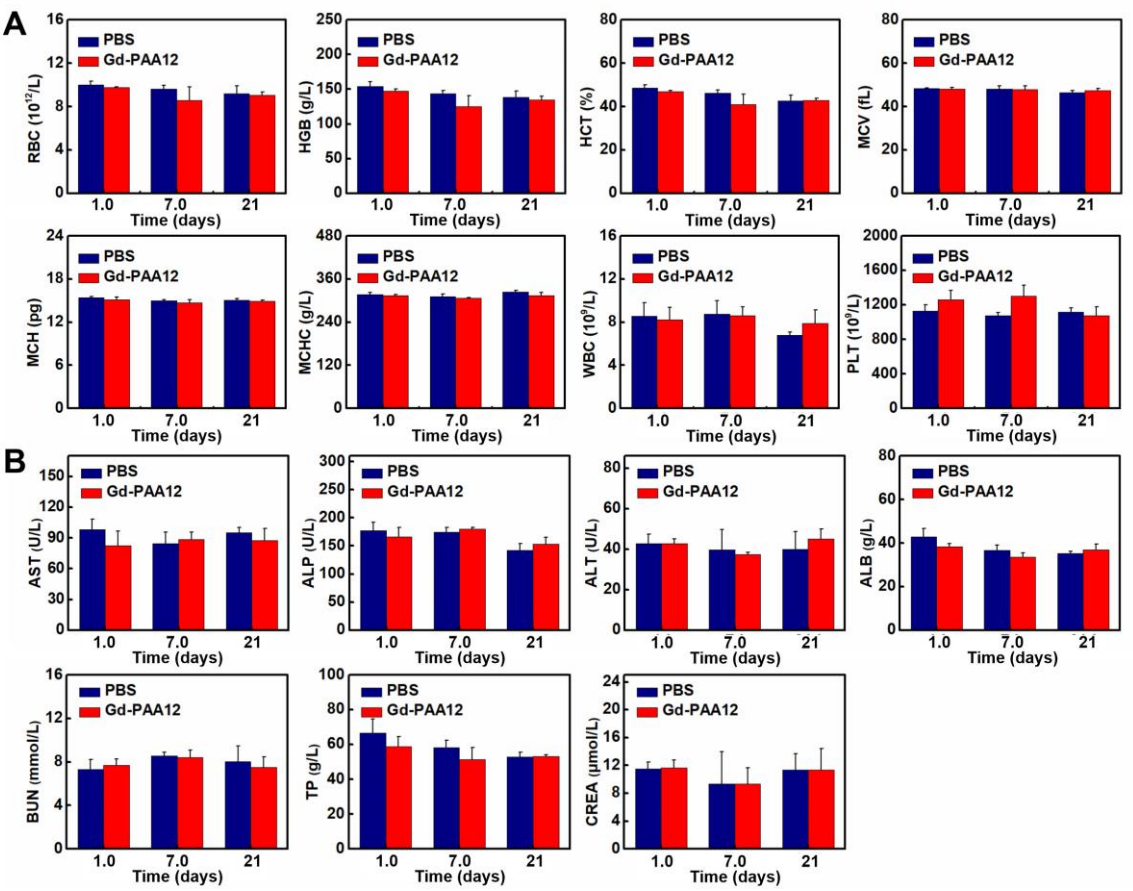


**Figure S14.** Blood routine analyses of heathy mice (A), or assessment of hepatic and renal functions for healthy mice by biochemical analyses (B) at day 1.0, 7.0, or 21 post-injection (*i.v.*) of PBS, or Gd-PAA12 ($n$ = 3). The blood routine analyses include the following indicators: red blood cells (RBC), hemoglobin (HGB), hematocrit (HCT), mean corpuscular volume (MCV), mean corpuscular haemoglobin (MCH), mean corpuscular haemoglobin concentration (MCHC), white blood cells (WBC) and platelets (PLT). Hepatic function indicators mainly include aspartate aminotransferase (AST), alkaline phosphatase (ALP), alanine aminotransferase (ALT), and albumin (ALB). Renal function indicators include blood urea (BUN), total protein (TP), and creatinine (CREA). Gd dosage = 5.0 mg/kg.

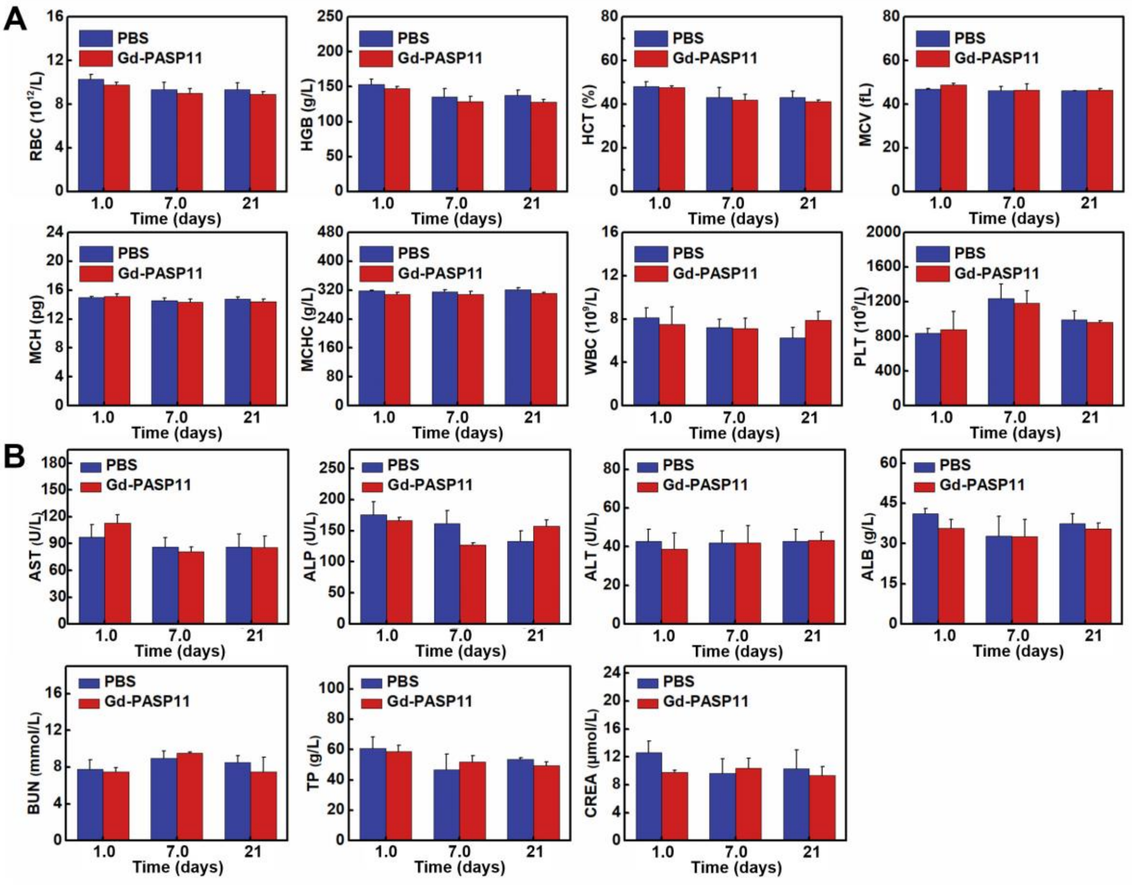


**Figure S15.** Blood routine and biochemical analyses of heathy mice. Blood routine analyses of heathy mice (A), or assessment of hepatic and renal functions for healthy mice by biochemical analyses (B) at day 1.0, 7.0, or 21 post-injection (*i.v.*) of PBS, or Gd-PASP11 (*n* = 3). Gd dosage = 5.0 mg/kg.

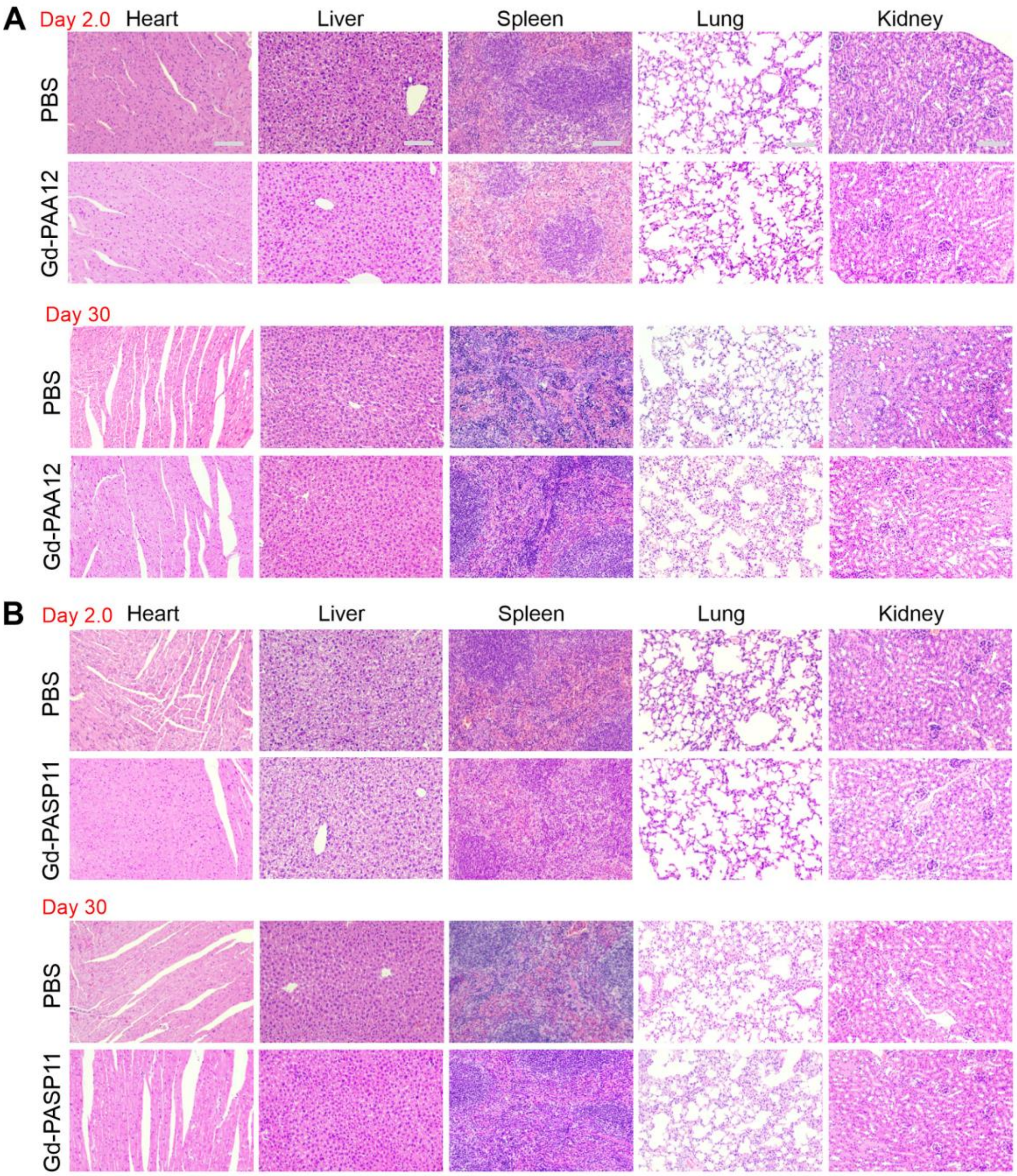


**Figure S16.** Histological analyses of main organs (H&E staining) obtained from healthy mice at day 2.0 or 30 post-injection (*i.v.*) of PBS, Gd-PAA12 (A), or Gd-PASP11 (B). Gd dosage = 10.0 mg/kg. Scale bar 100 μm.

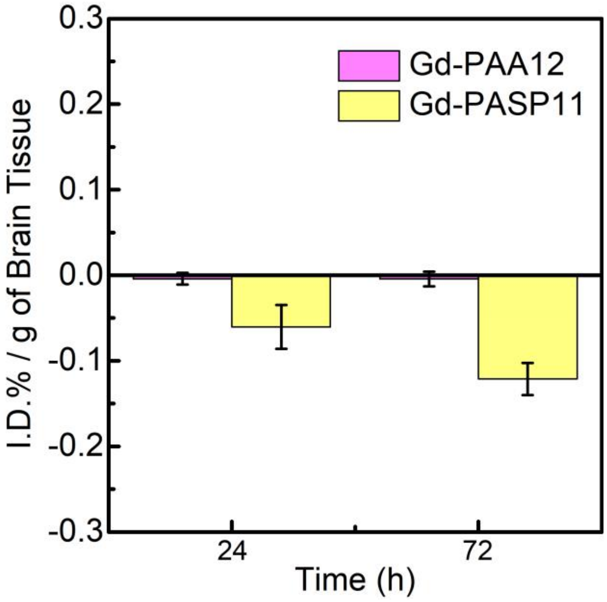


**Figure S17.** Gadolinium content of brain tissues at 24 h and 72 h. Gd dosage = 5.0 mg/kg.

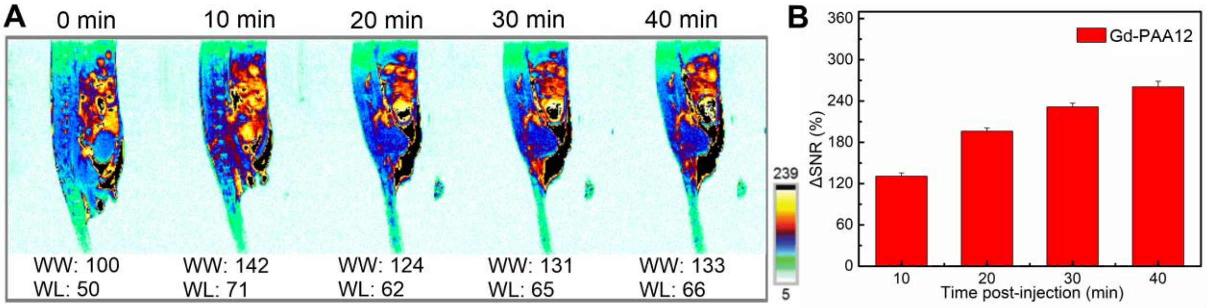


**Figure S18.** $T_1$-weighted MR images (A), and ΔSNR (B) of the bladders from 4T1 tumor-bearing mice showing evidence of renal excretion post-injection of Gd-PAA12. Slice orient: sagittal. Gd dosage = 5.0 mg/kg.

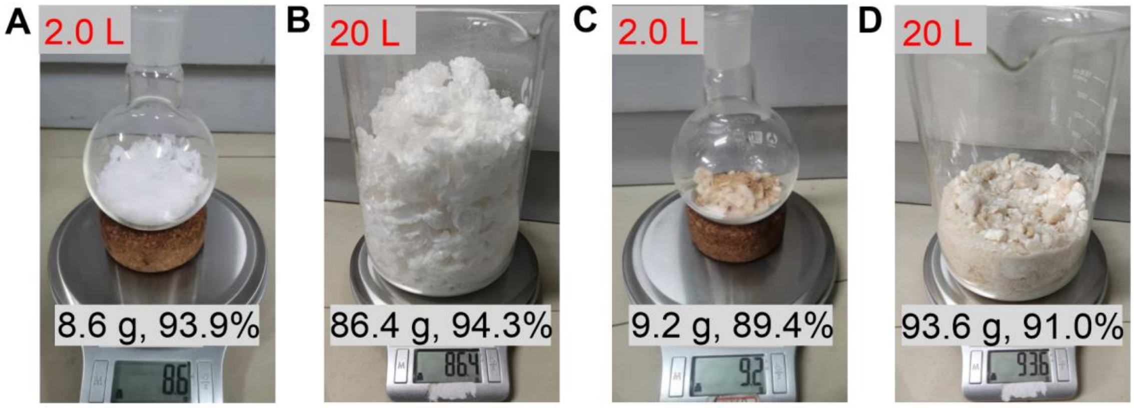


**Figure S19.** Weight and yield of the OGMC Gd-PAA12 (A, B), or Gd-PASP11 (C, D) synthesized in 2.0 L (A, C) or 20 L (B, D) of reactors after freeze-drying.

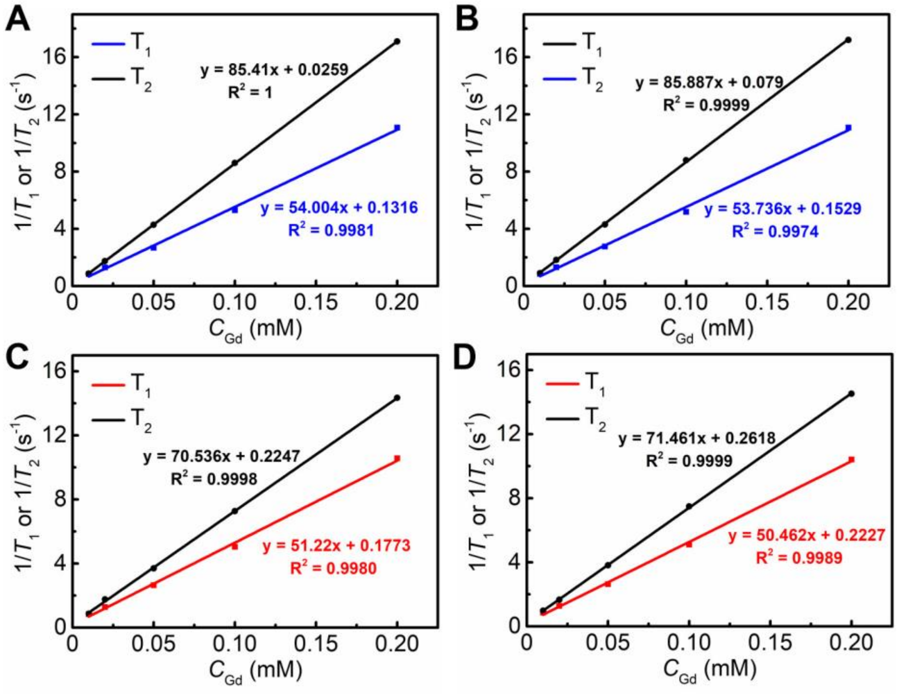


**Figure S20.** $T_1$ relaxation rate ($1/T_1$), or $T_2$ relaxation rate ($1/T_2$) plotted as a function of $C_{Gd}$ for Gd-PAA12 (A, B) or Gd-PASP11 (C, D) synthesized in 2.0 L (A, C), or 20 L (B, D) of reactors. Magnetic field = 3.0 T.

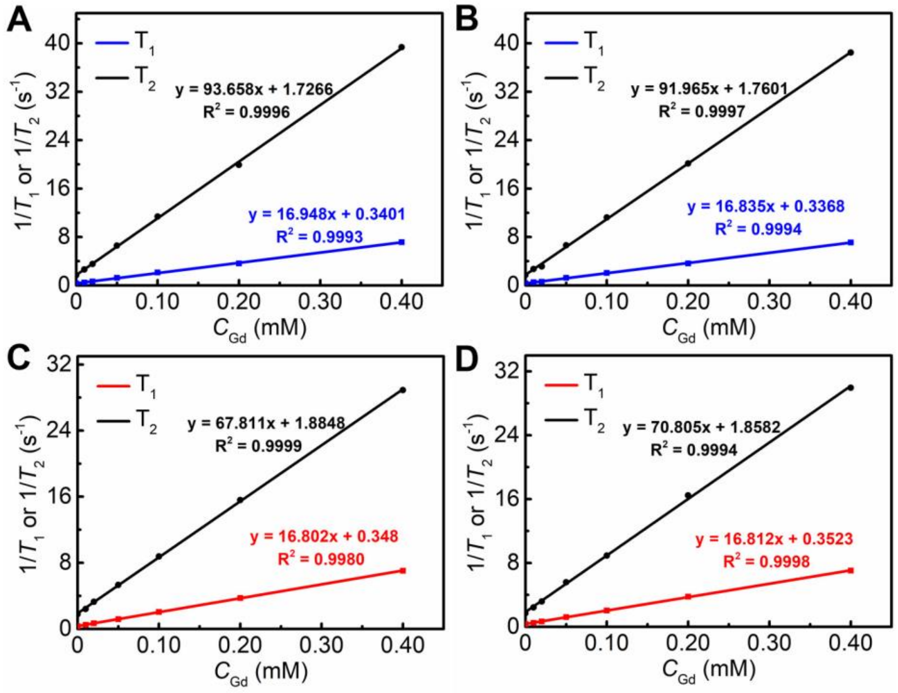


**Figure S21.** $T_1$ relaxation rate ($1/T_1$), or $T_2$ relaxation rate ($1/T_2$) plotted as a function of $C_{Gd}$ for Gd-PAA12 (A, B) or Gd-PASP11 (C, D) synthesized in 2.0 L (A, C), or 20 L (B, D) of reactors. Magnetic field = 7.0 T.
